# Multi-omics investigation of metabolic dysregulation in depression: integrating metabolomics, weighted gene co-expression network analysis, and mendelian randomization

**DOI:** 10.3389/fpsyt.2025.1627020

**Published:** 2025-08-12

**Authors:** Wu Qianhao, Zhang Jinwen, Miao Jingjie, Chen Xiaoyu, Zhao Yangfei, Yao Wenxiu, Jiang Xu, Wang Xiaojun, Han Peipei, Guo Qi

**Affiliations:** ^1^ Graduate School, Shanghai University of Traditional Chinese Medicine, Shanghai, China; ^2^ College of Rehabilitation Sciences, Shanghai University of Medicine and Health Sciences, Shanghai, China; ^3^ Science and Education Department, Shanghai Health Rehabilitation Hospital, Shanghai, China

**Keywords:** depression, metabolic diseases, untargeted metabolomics, Mendelian randomization, community-dwelling elderly

## Abstract

**Background:**

The etiology of depressive disorder, the leading cause of global mental disability, is characterized by systemic metabolic dysregulation. However, the causal metabolites and their mechanistic networks remain elusive.

**Methods:**

We combined untargeted LC/GC-MS metabolomics (N=98 Chinese elderly), weighted gene co-expression network analysis (WGCNA), and two-sample Mendelian randomization (MR) using GWAS data (59,333 depression cases with 434,831 controls) to identify depression-associated metabolites and pathways.

**Results:**

LC/GC-MS analysis identified 1,458 metabolites, with 84 differentially expressed in depression (VIP>1.5, p<0.05). WGCNA revealed a turquoise module enriched in amino acid metabolism (MM>0.7, p<0.05), while MR analysis confirmed 35 causal metabolites, including cysteine-alanine ratio (β=0.18, p=0.003) and serine levels (β=−0.24, p=0.001). Multi-omics integration highlighted glycine/serine/threonine metabolism (Impact = 0.35) and one-carbon folate cycle as core dysregulated pathways. Alterations were characterized by serine deficiency and phosphoserine accumulation, potentially reflecting disturbances in DNA methylation processes. Furthermore, elevated cysteine levels indicated a compensatory response to oxidative stress, and disruptions in purine metabolism pointed to mitochondrial dysfunction, particularly impaired mitochondrial ATP synthesis.

**Conclusion:**

This study establishes a hierarchical metabolic framework for depression, prioritizing single-carbon metabolism and oxidative stress as central therapeutic targets. The findings emphasize methylation dysregulation and mitochondrial dysfunction in elderly depression, offering novel biomarkers for precision intervention.

## 1 Introduction

Depressive Disorder (DD) is a debilitating mental disorder with a global prevalence of over 350 million cases (1), exhibiting a lifetime prevalence ranging from 7.5 to 20.6%, with a notable vulnerability among community-dwelling older adults (2, 3). This disorder poses a significant global health challenge. A multitude of factors have been identified as contributing to the onset and progression of depression, with inflammatory responses, dysregulation of the hypothalamic-pituitary-adrenal axis, imbalances in the sympathetic and parasympathetic nervous systems, and platelet-activated endothelial dysfunction being of particular note among the biological mechanisms. Depression is therefore considered to be a systemic disorder, with potential biomarkers of depression being identified in many different biological systems (4–6). Despite the proposal of several hypotheses to explain the pathogenesis of depression, the underlying molecular mechanisms remain unclear. A more profound comprehension of the molecular underpinnings of depression is imperative for the prevention or reduction of the global burden of this significant public health problem.

Metabolites are the products of upstream gene and protein regulatory networks and are involved in a wide range of physiological and pathological conditions (7). Off-target metabolomics, with its ability to cover a wide range of metabolites, has the potential to reveal previously unrecognized pathomechanisms and is a powerful tool for the discovery of small molecule metabolic markers in systems biology, as well as becoming an ideal tool for the discovery of DD biomarkers (8). To date, hundreds of metabolomics studies have been conducted to investigate metabolite alterations in animal models of depression, thereby expanding the understanding of the physiopathology of depression (9, 10). However, different expression trends were observed in the High-fat (HF) diet model metabolites: this may be related to the presence of species differences resulting in different rates of some metabolic pathways and metabolism. The paucity of population-based clinical studies significantly contributes to the observed variability.

Metabolomics is an effective method of revealing disease-associated metabolite changes. However, it is not easy to comprehensively resolve the complex network relationships among metabolites and the underlying biological mechanisms. It is particularly limited in exploring the causal effects of metabolites on disease (11). It is important to understand whether different metabolites act as risk or protective factors for DD, since this has implications for predicting disease and aiding diagnosis through specific targeting approaches. Mendelian randomization (MR) analysis employs single nucleotide polymorphisms (SNPs), which occur randomly in human genes, as instrumental variables (12). This approach is analogous to the design of a randomized controlled trial in that it enhances the randomization of sample selection. By establishing a link between the exposure factor and the outcome variable through the instrumental variable, the method provides a more reliable proof of causality between them (13). Conversely, weighted correlation network analysis (WGCNA) is a systems biology approach that uncovers intermolecular synergistic relationships, enabling the effective identification of important metabolite modules and aiding in the discovery of potential metabolites that have yet to be explored in metabolomics (14).

In this study, a comprehensive integration of non-targeted metabolomics, WGCNA, and MR approaches was performed to explore the mechanisms of metabolic dysregulation in depression and construct a framework for classifying metabolic pathways based on multi-omics data in depression. The study identified 1,458 metabolites in the blood of depressed (N = 98) and healthy Chinese populations and identified synergistic modules between metabolites. The study then screened for differential metabolite modules and potential key metabolites that were significantly associated with depression. Furthermore, the associations of 1400 blood metabolites and metabolite ratios between the depressed population (N = 59,333) cases and the healthy population (N = 434,831) were verified by MR analysis. Integration of metabolic pathway analysis was achieved by integrating highly associated metabolites identified by metabolomics, WGCNA, and MR methods. The objective of this study is to thoroughly examine the potential biological mechanisms of blood metabolites in depressed patients and non-depressed populations, to provide a new scientific basis for unraveling the metabolic mechanisms of depression, and to provide a theoretical basis for the discovery of metabolic pathways and the development of intervention targets.

## 2 Materials and methods

An overview of the study design and methodology is presented in **Supplementary Figure S1**.

### 2.1 Description of the sample

The present study employed a cross-sectional design, encompassing a population of community-dwelling older adults aged ≥65 years in Shanghai (n = 379). Informed consent was obtained from all participants who underwent a comprehensive geriatric assessment at a local community hospital, completed a standardized face-to-face interview, and provided a morning fasting venous blood sample. The Shanghai Medical College of Health Sciences Ethics Committee approved the study protocol. It was conducted strictly with the ethical guidelines of the Declaration of Helsinki. Informed consent was obtained from all subjects before they participated in the study (15, 16).

The questionnaire collection contained sociodemographic variables, including age and gender, and lifestyle factors such as smoking, alcohol consumption and daily activity level, the latter of which was assessed through the short form of the International Physical Activity Questionnaire (IPAQ). Health information covered body mass index (BMI), chronic diseases (e.g., diabetes, hypertension, hyperlipidemia, stroke and heart disease), substance use and cognitive function, the latter of which was assessed by the Brief Mental State Examination (MMSE). Further details about the questionnaire can be found in our previous study (17).

Participants were excluded if they (1) did not complete the questionnaire (n = 8), (2) were taking antidepressant medication (n = 2), or (3) lacked a blood sample (n = 1), resulting in a final sample size of 368 cases. A power analysis was conducted using GPower 3.1.9.7 software (https://www.psychologie.hhu.de/) to determine the appropriate sample size for this study. With an assumed effect size of 0.5 (medium effect), a significance level of 0.05, and a target statistical power of 0.80, the analysis indicated that a sample size of 49 participants per group was required, yielding a total sample of 98 participants (49 in the depressed group (DD group) and 49 in the non-depressed group (ND group)). This sample size was deemed adequate to ensure sufficient statistical power for detecting significant differences in metabolite levels between the two groups, thereby supporting the investigation of metabolic variations between depressed and non-depressed individuals. Forty-nine patients with confirmed depression and 49 non-depressed controls were selected from the remaining sample using a 1:1 frequency matching method, with matching variables including age (± 3 years) and gender.

### 2.2 Measurements of depression

The severity of depression was evaluated using the 30-item Geriatric Depression Scale (GDS) (18). On this scale, a score of 1 was allocated for a positive response to questions 2-4, 6, 8, 10-14, 16-18, 20, 22-26, and 28, and a score of 1 for a ‘no’ answer to questions 1, 5, 7, 9, 15, 19, 21, 27, 29, and 30. A mark was awarded for a ‘no’ answer to the aforementioned questions. According to the international consensus, a total score of >10 was defined as clinically significant depression (19).

### 2.3 Metabolomics analysis methods

Plasma samples were collected from the subjects in the morning fasting state using EDTA anticoagulation tubes. The plasma was separated by centrifugation at 1500×g for 15 min at 4°C and then immediately frozen in an ultra-low temperature refrigerator at -80°C. 150 μL of plasma was added to a pre-cooled methanol/acetonitrile mixture (2:1, v/v, containing 2-chlorophenylalanine internal standard, 0.3 mg/mL), vortexed. The mixture was then vortexed for one minute, followed by sonication for 10 minutes. Thereafter, the mixture was left to stand for 30 minutes before undergoing centrifugation at a speed of 13,000 rpm at a temperature of 4°C for 10 minutes. This process was intended to remove any precipitated proteins. The resultant upper layer was then subjected to freeze-drying under vacuum, which was re-dissolved in a methanol/water mixture (in a volume ratio 1:4). Thereafter, the solution was filtered through a 0.22 μm organic filter membrane. Liquid chromatography-mass spectrometry (LC-MS) analysis was performed on a Waters UPLC I-Class system coupled with a VION IMS QTOF high-resolution mass spectrometer (Waters Corp., Milford, USA), and the chromatographic separation was achieved using a BEH C18 column (2.1 × 100 mm, 1.7 μm) with mobile phases of 0.1% formic acid aqueous solution (phase A) and acetonitrile (phase B). The gradient elution procedure transitioned from 5% to 95% B over 0–12 min.

Gas chromatography-mass spectrometry (GC-MS) analysis was performed on an Agilent 7890B gas chromatograph coupled with a 5977A mass spectrometry detector (Agilent Technologies, Inc., CA, USA). The column used was a DB-5MS capillary column (30 m×0.25 mm ×0.25 μm). A total of 150 μl of plasma was added to an Eppendorf tube with 20 μl of 2-chlorophenylalanine (0.3 mg/ml) dissolved in methanol as an internal standard and vortexed for 10 s. Then, 450 μl of an ice-cold mixture of methanol/acetonitrile (2/1, v/v) to remove the protein was added to the tube and vortexed for 30 s. The mixture was extracted by ultrasonication in an ice water bath for 10 min, stored for 30 min (−20°C), and centrifuged at 4°C for 10 min (13,000 rpm). Two hundred milliliters of supernatant was placed into a new glass bottle, dried in a freeze concentration centrifuge and added to 80 μL of 15 mg/mL methoxylamine hydrochloride in pyridine. The resultant mixture was vortexed for 2 min and incubated at 37°C for 90 min. Then, 50 μL of BSTFA (with 1% TMCS) and 20 μL of n-hexane were added into the bottle, and the bottle was vortexed violently for 2 min and derivatized at 70°C for 60 min. The samples were placed at room temperature for 30 min before GC–MS. The internal standard peaks’ relative standard deviation (RSD) was controlled within 15%, consistent with our previous study procedure (15).

The LC–MS data were analyzed using Proggenesis Qi software version 2.3 (Nonlinear Dynamics, Newcastle, UK). Initially, the software was used to perform data mining, advanced alignment, peak picking, normalization, and retention time (RT) correction. The resulting characteristic matrix includes information on the mass-to-charge ratio (m/z), RT, and peak intensities. In the chromatographic column, ion peaks are separated, and the resulting RT serves as a reference parameter for the preliminary identification of metabolites. First-level mass spectrometry (MS1) provides the accurate m/z of metabolites, which can be used to infer their molecular formula, with isotope distribution aiding in the inference. Second-level mass spectrometry (MS/MS) analyzes the fragmentation of the parent ion (the molecular ion) to generate fragment ions, and the analysis of the fragmentation pattern helps infer structural features. Subsequently, the identification of metabolites was based on precise m/z, secondary fragments, and isotope distribution, using the Human Metabolome Database (HMDB) (http://www.hmdb.ca/), LipidMaps (version 2.3) (http://www.lipidmaps.org/), METLIN (http://metlin.scripps.edu/), and self-built databases (EMDB) for qualitative analysis. Main parameters of 5 ppm precursor tolerance, 10 ppm product tolerance, and 5% product ion threshold were applied. Compounds with resulting scores below 36 (out of 60) points were also deemed to be inaccurate and removed.

The GC–MS data used the software MS-DIAL (version 2.74) for peak detection, peak identification, characterization, peak alignment, wave filtering, etc. The ionization method used in GC-MS was electron impact ionization (EI), which generates highly consistent fragment ions. MS1 provides abundant structural information, which can be matched with the LUG database (Untargeted database of GC–MS rom Lumingbio). Compounds with resulting scores below 50 (out of 100) points were also deemed to be inaccurate and removed. The raw data matrix was obtained from the raw data with a three-dimensional dataset, including sample information, the name of the peak of each substance, retention time, retention index, mass-to-charge ratio, and signal intensity, after alignment with the Statistical Compare component. The internal standards with RSD>0.3 were used to segment and normalize all peak signal intensities in each sample, and the segmented and normalized results were removed redundancy and merged peak to obtain the data matrix.

Between-group differences were assessed using the OPLS-DA model in combination with the permutation test (200 times), with the following screening criteria: VIP>1.0 and p<0.05 (two-sided t-test). The Kyoto Encyclopedia of Genes and Genomes (KEGG) database (http://www.kegg.jp/kegg/pathway.html) was used for KEGG pathway enrichment analysis (Fisher’s exact test, FDR correction) to reveal significant metabolic pathways. Finally, the differential metabolites obtained from the LC-MS and GC-MS analyses were taken and pooled into a pool of highly related metabolites, and pathway enrichment analysis was performed with pathway analysis.

### 2.4 Weighted gene co-expression network analysis

In this study, metabolomics data were systematically integrated by WGCNA to identify metabolite modules significantly associated with depression and to explore key metabolites further. The analysis utilized data derived from a differential metabolite matrix obtained using LC/GC-MS. The analysis process comprised four principal steps: network construction, module identification, association analysis of modules with phenotypic features, and network visualization (20). The WGCNA analysis was implemented in the R software environment using the WGCNA package (version 1.73) for processing metabolomics datasets. To reduce the potential heterogeneity in the results of LC and GC analyses, WGCNA analysis was performed in this study on the results of the two analytical methods separately. The construction of co-expression networks was achieved by selecting metabolite data from the metabolomics dataset corresponding to the top 50% of variation (14).

The dynamic shear tree method was employed during the module merging process, with a threshold set at 0.25. The network was subjected to soft-threshold screening to ensure it met the scale-free topological properties (scale-free fit index R² ≥ 0.60). The final soft thresholds for LC-MS and GC-MS data were determined to be β = 5 (R² = 0.7) and β = 3 (R² = 0.6), respectively. Other network construction criteria included the minimum number of metabolites per module, which was set at 10. The WGCNA analysis defined the co-expression relationship of metabolites within a module by calculating Module Membership (MM) and Gene Significance (GS). The higher the MM value, the stronger the co-expression correlation of the metabolite within the module, while a higher GS value indicates that the metabolite is more biologically significant in the cluster module. Subsequent screening of key metabolites was then conducted based on the significant correlation between the module as a whole and the phenotype (MM correlation p-value < 0.05). The metabolites of highly connected nodes within the modules were then identified based on MM values, and these were thus identified as potential latent key metabolites. The identification of significant potential key metabolites was achieved by examining the corrected GS values and their p-values for phenotypic correlation (MM > 0.7, GS > 0.2, p < 0.05). The potential key metabolites generated from both analyses were pooled for pathway enrichment and analysis.

### 2.5 Mendelian randomization of data sources

The metabolite genome-wide association study (GWAS) data in this study were obtained from the GWAS Catalog public database (https://www.ebi.ac.uk/gwas/) (accession ID: GCST90201021-GCST90204063), covering 1091 plasma metabolites and 309 metabolite ratios. Genetic instruments for metabolites were derived from a large-scale GWAS conducted on 8,299 unrelated European participants from the Canadian Longitudinal Study of Aging (CLSA). Participants underwent genome-wide genotyping using the Affymetrix Axiom platform, with imputation performed through the Trans-Omics for Precision Medicine (TOPMed) program. The sample overlap with the depression GWAS from the FinnGen study version 12 was minimal, ensuring no significant overlap between the target metabolites and depression-associated SNPs. After rigorous quality control, single nucleotide polymorphisms (SNPs) with a minor allele frequency (MAF) greater than 0.1%, an imputation quality score above 0.3, and a missing rate below 0.1% were retained, resulting in approximately 15.4 million SNPs. Plasma metabolite levels were quantified using the Metabolon HD4 UPLC-MS/MS platform, with 1,091 metabolites retained after excluding those with more than 50% missing data. Of these, 850 metabolites were classified into eight metabolic super-pathways: lipids, amino acids, xenobiotics, nucleotides, cofactors and vitamins, carbohydrates, peptides, and energy, while 241 were categorized as “unknown” or “partially characterized.” The data underwent log transformation, standardization, and removal of outliers beyond three standard deviations. Metabolite ratios were constructed from 309 pairs of metabolites sharing enzymes or transport proteins, as identified in the Human Metabolome Database (HMDB). The depression GWAS data were obtained from the FinnGen study version 12 (finngen_R12_F5_DEPRESSIO) in Finland, containing genotype data from 59,333 depressed patients and 434,831 healthy controls (total number of SNPs = 20,112,636). The raw VCF files were then filtered by PLINK v1.9 for quality control (MAF > 0.01, HWE p > 1 × 10^-6, deletion rate 0.05). SNPs significantly associated with the target metabolites were finally extracted for subsequent analyses.

### 2.6 Choice of instrumental variables and chain imbalance adjustment

In the MR analysis, the selection of instrumental variables was based on the following three core assumptions: (1) Strong correlation of instrumental variables: for each metabolite, a genome-wide significance threshold (p < 1 × 10^–5) was set to select SNPs that were strongly correlated with the metabolite. This ensured that the relationship of the selected SNPs with the target metabolite was statistically significant. The F-statistics for the selected SNPs were calculated and confirmed to be above the threshold of 10, indicating strong instrument relevance. (2) Linkage disequilibrium (LD): Following the extraction of significant SNPs, an LD analysis was performed to assess the correlation between SNPs. The existence of interlocking disequilibrium between the pair of SNPs was considered to have occurred if the coefficient of linkage disequilibrium (r²) was less than 0.001 and the distance between the SNPs was less than 10,000 bases (kb). The analysis of linkage disequilibrium ensured that each instrumental variable independently affected a specific metabolite and reduced potential pleiotropy. (3) Independence of instrumental variables: To avoid interdependence between instrumental variables, SNPs associated with depression were excluded from the analyses, thus reducing the possible direct relationship between instrumental variables and outcomes (21). Furthermore, Steiger filtering was performed to ensure the correct direction of causality between SNPs and metabolites, confirming that the instruments were appropriately aligned with the exposure and not the outcome. Although all instrumental variables met the above assumptions, non-associated SNPs may still influence the occurrence of depression. Therefore, linkage disequilibrium score (LDSC) regression analyses were performed to calculate multiple validity and correct for potential bias (12).

### 2.7 Mendelian randomization analysis

In this study, the causal relationship between metabolites and depression was assessed using the standard inverse variance weighting (IVW) method. This is a commonly used and robust technique for estimating causal effects, which accurately estimates the causal effect of exposure (metabolites) on the outcome (depression) when the instrumental variables satisfy all the core assumptions (22). To further validate the robustness of the results, the MR-Egger and weighted median (WM) methods were used as secondary assessment tools. These methods can provide complementary results to the IVW methods and help identify possible biases. Mendelian randomization analyses were conducted in the R environment using the TwoSampleMR package (version 0.6.16) and the Mendelian Randomization package (version 0.10.0), ensuring consistent and reproducible implementation of the analytical pipeline. After KEGG database matching, all metabolites with possible causality were included in the high-association metabolite pool.

### 2.8 Highly associated metabolite pool building

This study constructed a high-confidence metabolite pool by integrating significant metabolites from multiple methods. The metabolites in question were those with VIP > 1.0 and p < 0.05, as determined by the LC/GC-MS platform. The identification of pivotal metabolites was achieved through WGCNA, which sets MM greater than 0.7 and GS greater than 0.2 with a P-value less than 0.05 as the screening criteria. Furthermore, causal metabolites with a P-value less than 0.05 were identified as associative metabolites using the IVW method in MR analysis. Metabolites identified by all methods were assigned no weights and were directly merged according to the metabolites identified by each method. This was done to ensure that the metabolite pool contained support from multidimensional evidence (23).

### 2.9 Pathway enrichment analysis and pathway analysis methods

Metabolite pathway enrichment and pathway analysis were performed using the MetaboAnalyst 5.0 platform (https://www.metaboanalyst.ca). All metabolites were matched by KEGG ID and subjected to pathway enrichment and pathway analysis based on the metabolic pathways included in the KEGG database. The selection of metabolic pathways as significantly enriched pathways for pathway enrichment analysis was made based on p-values less than 0.05. The same p-value threshold was applied to select significant pathways for pathway analysis. The pathway enrichment results were visualized using the Graph and ggplot2 R packages.

### 2.10 Criteria for the classification of pathways

LC/GC-MS analyzed the differential metabolites, WGCNA analyzed the key metabolites, the metabolites included in positive results by MR analysis, and the pools of highly correlated metabolites were subjected to pathway enrichment analysis and pathway analysis, respectively. These were then labeled as Class I, II, and III pathways based on the following Classification criteria, which were derived from the comprehensive analysis using MetaboAnalyst. The custom Class I/II/III system, particularly the impact threshold, was informed by MetaboAnalyst’s analysis methodology. The following Classification criteria identified the closely related metabolic pathways (9, 10).

Class I pathway: Impact ≥ 0.25; significantly enriched and expressed by at least two methods in LC/GC-MS, WGCNA, and MR analyses (P < 0.05), and significantly enriched in metabolite pooling analyses with consistent directionality;

Class II pathway: 0.1 ≤ Impact ≤ 0.25; significant (P < 0.05) by LC/GC-MS or MR; partially consistent metabolite trends;

Class III pathway: Impact ≤0.1, significant (p < 0.05), or contradictory metabolite trends that suggest an association. In this instance, further validation is required.

### 2.11 Statistical analysis methods

The initial investigation into the baseline sociodemographic and health-related characteristics was conducted utilizing SPSS version 25.0 (SPSS Incorporated, Chicago, IL, USA), with a statistical significance threshold of p< 0.05. A subsequent comparison was made between DD and ND, employing independent t-tests for numerical variables and chi-square tests for categorical variables. Data that conformed to a normal distribution was expressed as the mean ± standard deviation (SD), while categorical variables were presented as proportions. All analyses were conducted in the R v4.3.1 environment, and MR analyses were performed using dedicated packages such as TwoSampleMR and Mendelian Randomization.

## 3 Result

### 3.1 Characteristics of the study population

According to the exclusion criteria, 11 subjects were eliminated from the study, and the remaining 368 subjects were included in the experiment. Of the 368 subjects included in the experiment, 49 were diagnosed with DD according to the diagnostic criteria.319 patients without depression were selected, and 49 were matched by age and gender with the DD group as ND. As demonstrated in **Table 1**, there was no significant difference between DD and ND regarding socio-demographics, lifestyle and health status (p < 0.05). A statistically significant difference was observed in GDS scores between the two groups (p < 0.001).

**Table 1:** Baseline sociodemographic variables of the included population (N=98).

Characteristic	DD (n=49)	ND (n=49)	p Value
Age (years)	72.10 ± 5.12	73.47 ± 4.49	0.163
Sex (%)			0.671
Male	36.7	32.7	
Female	63.3	67.3	
Smoking (%)			0.727
No	91.8	89.8	
Yes	8.2	10.2	
Drinking (%)			0.133
No	85.7	73.5	
Yes	14.3	26.5	
BMI (kg/m²)	23.64 ± 3.59	24.27 ± 3.89	0.412
IPAQ (Met-min/wk)	5977.30 ± 5977.31	6385.78 ± 5391.08	0.712
Total cholesterol (mmol/L)	5.23 ± 0.94	5.29 ± 1.06	0.748
Triglycerides (mmol/L)	1.32 ± 0.70	1.27 ± 0.72	0.732
HDL (mmol/L)	1.41 ± 0.77	1.51 ± 0.39	0.129
LDL (mmol/L)	3.38 ± 0.78	3.39 ± 0.99	0.919
Number of diseases			
Diabetes (%)			0.316
No	75.5	83.7	
Yes	24.5	16.3	
Hypertension (%)			0.667
No	30.6	34.7	
Yes	69.4	65.3	
Hyperlipidemia (%)			0.505
No	87.8	91.8	
Yes	12.2	8.2	
Stroke			0.277
No	63.3	73.5	
Yes	36.7	26.5	
Heart disease (%)			0.671
No	63.3	73.5	
Yes	36.7	26.5	
MMSE	24.69 ± 4.67	23.94 ± 4.80	0.432
GDS score	14.71 ± 3.57	4.90 ± 2.37	<0.001

*DD, depression; ND, non-depression; BMI, body mass index; IPAQ, international physical activity questionnaire; HDL, high-density lipoprotein; LDL, low-density lipoprotein; MMSE, Mini-mental State Examination; GDS, score Geriatric Depression Scale score.

### 3.2 Non-targeted LC/GC-MS results

**Figure 1:**
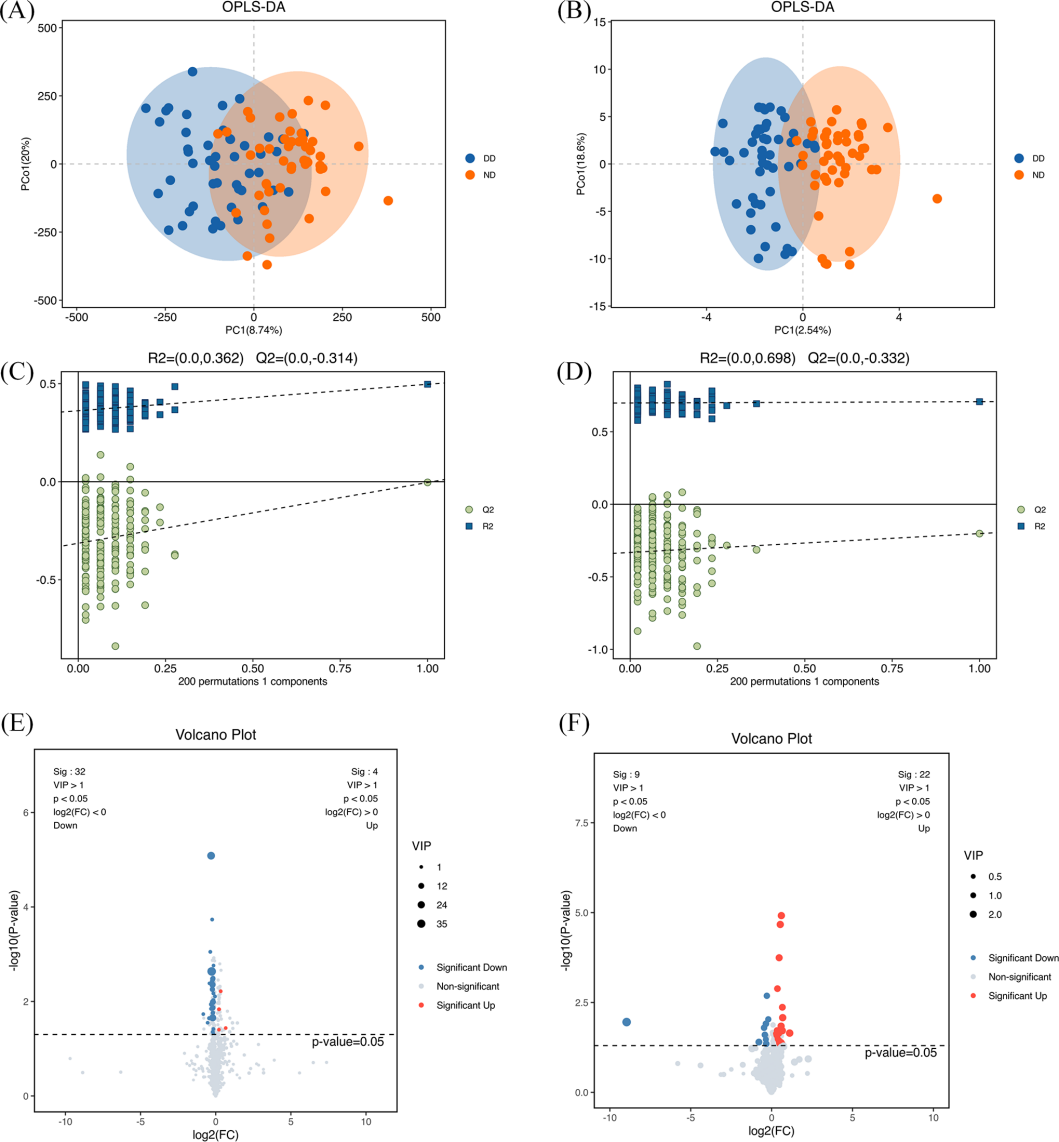
Multivariate statistical analysis and differential metabolite profiling of serum metabolites based on LC/GC-MS analysis for the DD and ND groups.**(A)** OPLS-DA score plots based on LC-MS analysis show the separated DD and ND groups. **(B)** OPLS-DA score plots based on GC-MS analysis showing group separation. **(C)** Statistical validation of the LC-MS-based OPLS-DA model showing R² (0.362) and Q² (0.314). **(D)** Statistical validation of the GC-MS-based OPLS-DA model showing R² (0.662) and Q² (0.532). **(E)** LC-MS-based volcano plot showing differential metabolites between DD and ND groups. Blue dots represent metabolites with decreased levels, red dots represent metabolites with increased levels, and grey dots represent metabolites with no significant change. The area size of the dots reflects the VIP value. **(F)** The GC-MS-based volcano plot shows similar differential metabolites with color coding as described above. The two coordinate points on the plots **(A–D)** are relatively distant, indicating a significant difference between the two samples and vice versa. Elliptical regions represent 95% confidence intervals.

1,012 compounds were identified in plasma by LC-MS and 446 compounds by GC-MS (**Supplementary Tables S1**, **S2**). The differences in plasma metabolites between the two groups of samples were assessed using the OPLS-DA model, which demonstrated separation and minimal overlap between the two groups (**Figures 1A, B**). The model’s reliability was confirmed by 200 response permutation tests (**Figures 1C, D**). The first principal component of the OPLS-DA model was used to identify differential metabolites. The screening criteria were a VIP value >1.5 and a t-test p-value <0.05. This identified 65 metabolites by LC-MS and 19 by GC-MS. The 30 metabolites with the highest VIP values are shown in **Supplementary Table S2**. The volcano plot (**Figures 1E, F**) demonstrates the validity of the differential metabolites by displaying the p-values and fold change values.

**Figure 2:**
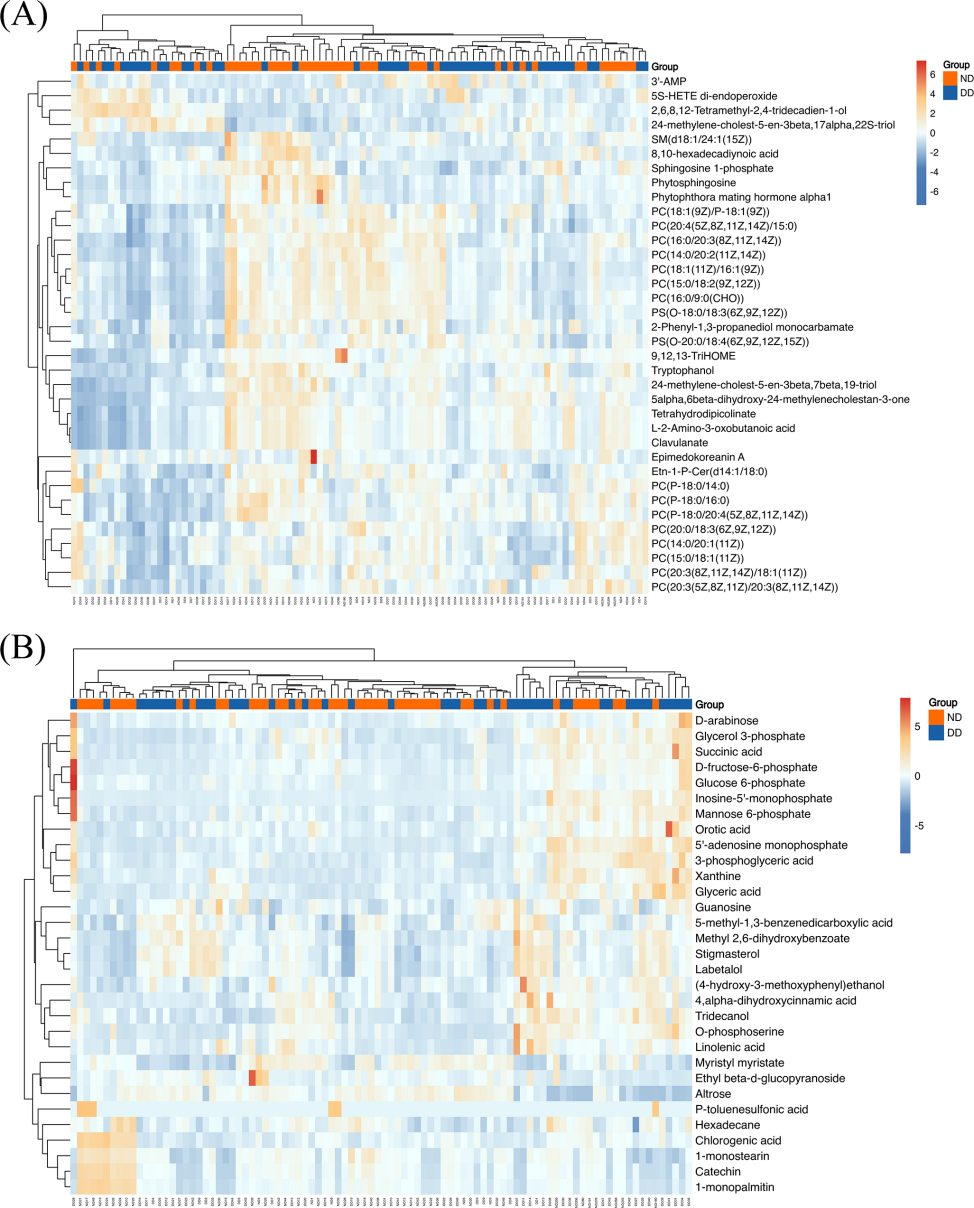
**(A)** LC-MS-based hierarchical clustering heatmap showing metabolite expression patterns for DD and ND groups. The color scale from blue to red represents increasing metabolite expression. **(B)** GC-MS-based hierarchical clustering heatmap showing similar clustering patterns as in **(A)**, with a color scale indicating expression levels from low (blue) to high (red).

**Figure 3:**
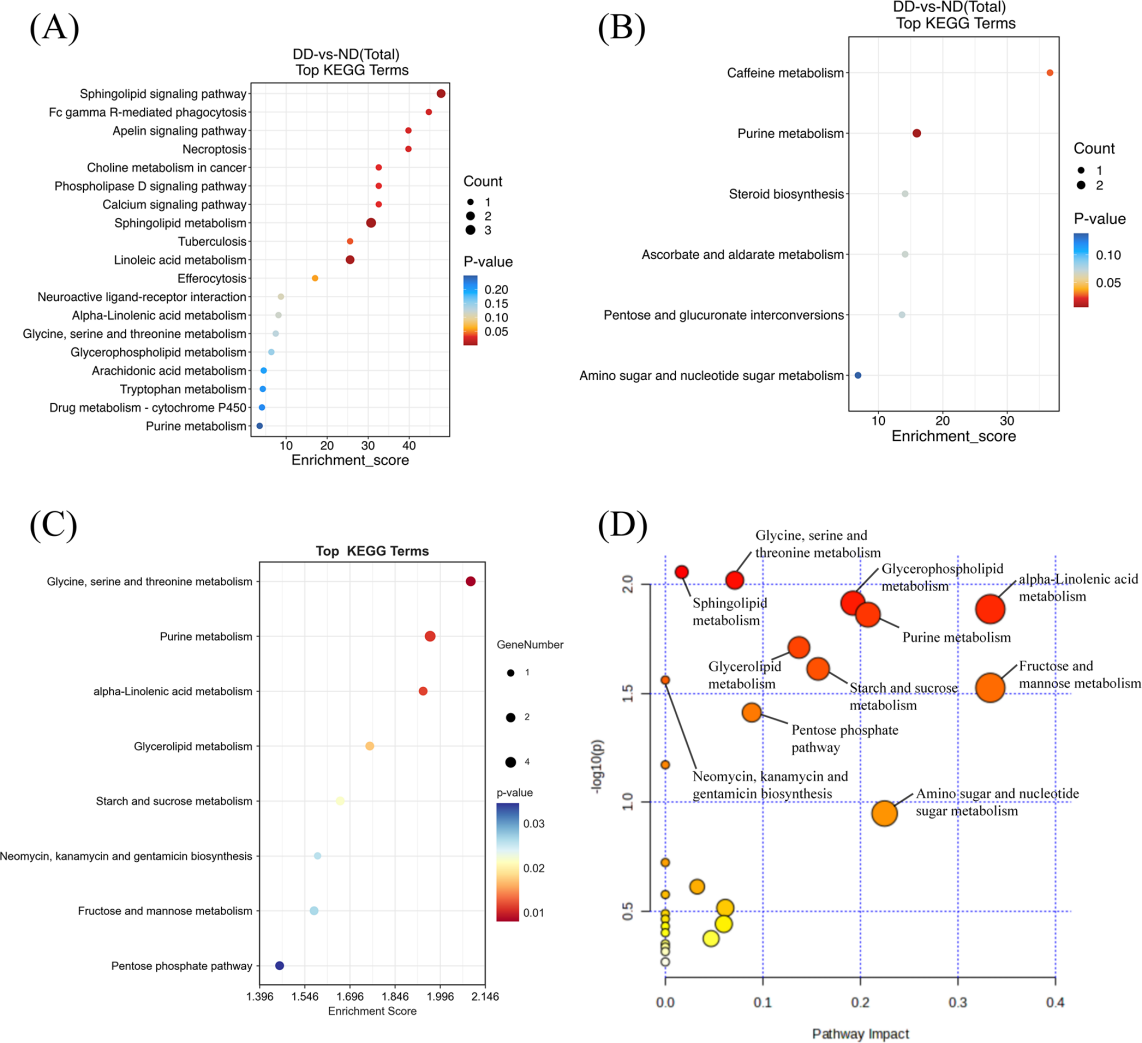
Metabolic pathway enrichment analysis of differentially expressed metabolites from LC-MS and GC-MS data.**(A)** Pathway enrichment analysis of metabolites from LC-MS data. Each bubble represents a distinct metabolic pathway, with the size of the bubble indicating the number of differential metabolites within that pathway. The color intensity of the bubble reflects the statistical significance (p-value) of the pathway, with red indicating higher significance. **(B)** Pathway enrichment analysis of metabolites from GC-MS data, showing similar representations of pathway significance and metabolite count. **(C)** Pathway enrichment analysis of combined LC-MS and GC-MS data, with bubble size corresponding to the number of differential metabolites and color indicating the pathway’s significance. **(D)** Pathway impact analysis for combined LC-MS and GC-MS data. Each point represents a metabolic pathway, with the X-axis indicating the pathway’s impact and the Y-axis indicating its statistical significance (p-value). More prominent points represent pathways with a higher contribution to the metabolic network, while smaller p-values indicate more statistically significant pathways.

Hierarchical clustering is employed to demonstrate the levels of these metabolites, with colors indicating higher (red) or lower (blue) levels and intensities reflecting the corresponding concentrations (**Figure 2**). KEGG enrichment analyses were performed for LC-MS, GC-MS, and LC/GC-MS corresponding to the differential metabolites, respectively (**Figure 3**). The results showed that amino acid, lipid, purine, and carbohydrate metabolism were the main enriched pathways for metabolic abnormalities in depressed patients. Among them, Glycine, Serine and Threonine Metabolism were significantly enriched in the LC-MS platform (P < 0.05), and Sphingolipid Metabolism was also enriched (P < 0.05). In addition, Purine Metabolism showed significant enrichment (P < 0.05), and GC-MS analysis showed that Caffeine Metabolism, Steroid Biosynthesis and Pentose and Glucuronate Interconversions were also significantly enriched (P < 0.05) (**Figure 3B**). After the combination of the LC-MS and GC-MS results, the enriched pathways were expanded to encompass One Carbon Pool by Folate, Starch and Sucrose Metabolism and Glycerophospholipid Metabolism in addition to an amino acid, lipid and nucleotide metabolism (P < 0.05) (**Figure 3C**).

The Pathway Impact Analysis further assessed the impact of different metabolic pathways on the overall metabolic network (**Figure 3D**). The results showed that neurotransmitter, purine, glutathione, and ceramide metabolism had a high degree of pathway impact (impact > 0.2). Of these, glycine, serine and threonine metabolism demonstrated the most significant impact (impact > 0.3), while purine metabolism and the pentose phosphate pathway exhibited impacts exceeding 0.2. Within the domain of lipid metabolism, ceramide metabolism and glycerophospholipid metabolism showed impacts that surpassed 0.25. The collective analysis of the metabolic pathway enriched results indicated that metabolic abnormalities in patients with depression were associated with various factors, including neurotransmitter synthesis, energy supply, membrane lipid metabolism and oxidative stress.

### 3.3 Key metabolic modules associated with WGCNA

This study constructed metabolic co-expression networks using WGCNA based on metabolite data from LC-MS and GC-MS platforms to identify key metabolic modules associated with depression and further resolve their functional characteristics. The scale-free property of the co-expression network was ensured by setting the optimal soft threshold at 5 (R² = 0.7, A) for LC-MS data and 3 (R² = 0.6, **Supplementary Figure S2A**) for GC-MS data. The application of cluster analysis to the LC-MS data resulted in the identification of multiple co-expression modules, with turquoise, blue, red, and yellow modules containing a higher number of metabolites (**Supplementary Figure S2C**). A similar observation was made in the GC-MS data, where the metabolite co-expression network also identified multiple modules, with turquoise and red modules being particularly enriched in metabolites. (**Supplementary Figure S3B**) Further analysis revealed that between ND and DD, the turquoise modules of the LC-MS data (P = 0.002) and the turquoise module of the GC-MS data (P = 0.04) were significantly correlated with the depression phenotype. (**Supplementary Figure S2D**, **Supplementary Figure S3D**) Module Membership was further analyzed in this study to determine the functional importance of key metabolic modules. (**Supplementary Figure S2B**) At the same time, a similar trend was observed in the cyan module of the GC-MS data (**Supplementary Figure S3C**). The results suggest that the turquoise module may contain important metabolites that regulate the development of depression and the metabolites contained in the turquoise module were screened with potential key metabolite criteria (MM > 0.7, GS > 0.2, p < 0.05) before further analysis and inclusion into the metabolite pool. The specific metabolites included are shown in **Supplementary Table S3**.

**Figure 4:**
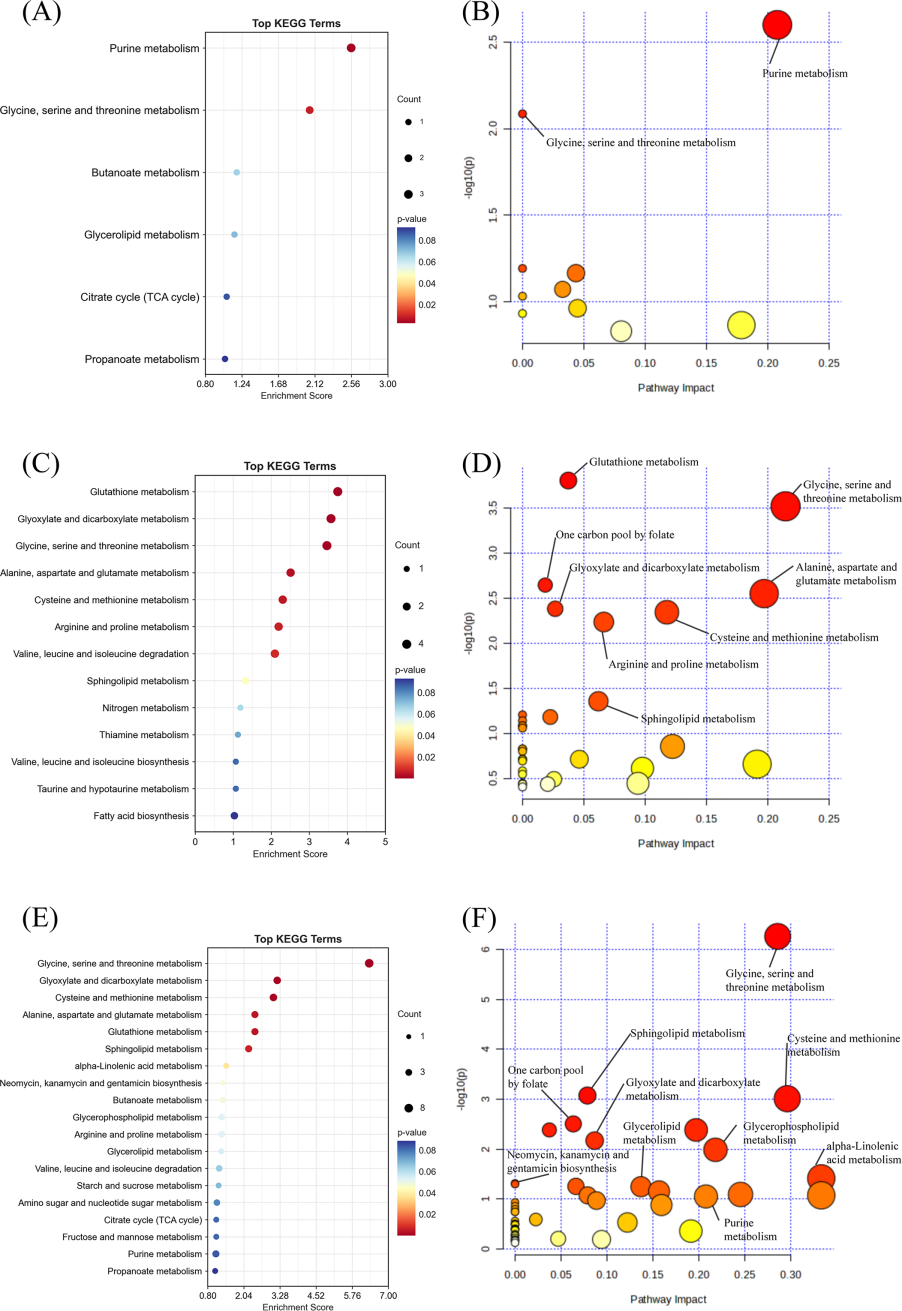
Pathway enrichment and pathway impact analysis. **(A)** Pathway enrichment analysis of WGCNA results from combined LC/GC-MS data. **(B)** Pathway impact analysis of WGCNA results from combined LC/GC-MS data. **(C)** Pathway enrichment analysis of positive metabolites from MR analysis. **(D)** Pathway impact analysis of positive metabolites from MR analysis. **(E)** Pathway enrichment analysis of metabolites from LC/GC-MS, WGCNA metabolites, and MR-positive metabolites. **(F)** Pathway impact analysis of metabolites from LC/GC-MS, WGCNA metabolites, and MR-positive metabolites.

KEGG enrichment analysis for pathway enrichment and pathway analysis demonstrated that multiple core metabolic pathways were significantly enriched (P < 0.05), including Purine Metabolism, Glycine, Serine and Threonine Metabolism, and Butanoate Metabolism. Furthermore, a higher impact was observed for Purine Metabolism in the Pathway Impact Analyses, suggesting a central role for this pathway in depression-related metabolic abnormalities (**Figures 4A, B**).

### 3.4 MR analysis results

**Figure 5:**
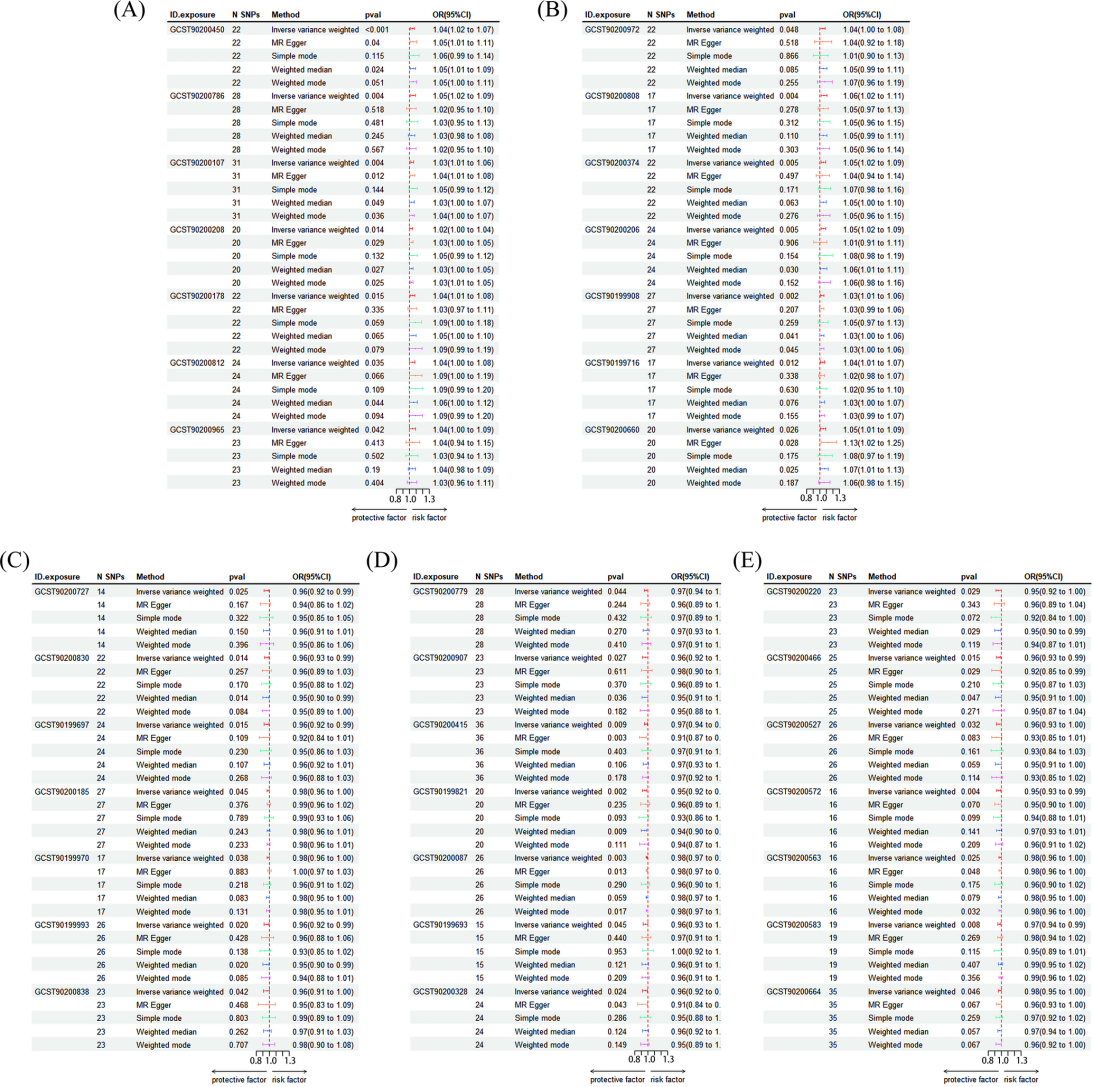
Risk forest plot of the results of MR Analysis. **(A)** GCST90200450: Caproate (6:0) levels; GCST90200786: Cysteine to alanine ratio; GCST90200107: Gamma-glutamyl-alpha-lysine levels; GCST90200208: Glucuronide of piperine metabolite C17H21NO3 (4) levels; GCST90200178: Glucuronide of C10H18O2 (7) levels; GCST90200812: Glutamate to kynurenine ratio; GCST90200965: Glutamate to 5-oxoproline ratio; **(B)** GCST90200972: Leucine to N-palmitoyl-sphingosine (d18:1 to 16:0) ratio; GCST90200808: Spermidine to choline ratio; GCST90200374: Sphinganine levels; GCST90200206: Sulfate of piperine metabolite C18H21NO3 (1) levels; GCST90199908: 2-o-methylascorbic acid levels; GCST90199716: 10-undecenoate (11:1n1) levels; GCST90200660: X-25957 levels; **(C)** GCST90200727: Adenosine 3’,5’-cyclic monophosphate (cAMP) to taurocholate ratio; GCST90200830: Cholate to adenosine 3’,5’-cyclic monophosphate (cAMP) ratio; GCST90199697: Dodecanedioate levels; GCST90200185: Gamma-glutamylcitrulline levels; GCST90199970: Octadecenedioylcarnitine (C18:1-DC) levels; GCST90199993: Phenylacetylcarnitine levels; GCST90200838: Phosphate to glutamate ratio; **(D)** GCST90200779: Pyruvate to N-acetylneuraminate ratio; GCST90200907: Retinol (Vitamin A) to linoleoyl-arachidonoyl-glycerol (18:2 to 20:4) ratio; GCST90200415: Serine levels; GCST90199821: 1-linoleoyl-GPI (18:2) levels; GCST90200087:2-methylserine levels; GCST90199693: 3-hydroxydecanoate levels; GCST90200328: 3-methoxytyrosine levels; **(E)** GCST90200220: 4-allylcatechol sulfate levels; GCST90200466: X-11632 levels; GCST90200527: X-13866 levels; GCST90200572: X-19299 levels; GCST90200563: X-21355 levels; GCST90200583: X-21807 levels; GCST90200664: X-25810 levels.

This study lists the names of 1,400 related blood metabolites and their respective ratios. (**Supplementary Table S4**) The details of all the included instrumental variables can be found in **Supplementary Table S5**. The IVW-based MR analysis identified 35 metabolites that were significantly associated with depression (p < 0.05). (**Supplementary Tables S6**, **S7**) Of these, 14 metabolites were positively associated with the risk of depression, while 21 metabolites were negatively associated with the risk (**Figure 5**). The confidence intervals for these causal estimates ranged from 95%CI. Sensitivity analyses demonstrated that the MR-Egger regression intercept test did not identify substantial pleiotropy, and the Weighted Median and Simple Mode results were broadly consistent with the IVW direction, thereby confirming the robustness of the causal relationships. The positively correlated metabolites were primarily associated with amino acids and their metabolites (e.g., the cysteine to alanine ratio, the glutamate to kynurenine ratio), lipid metabolism (e.g., the sphinganine levels, the leucine to N-palmitoyl-sphingosine ratio), and the antioxidant defense system (e.g., the 2-O-ethyl ascorbic acid levels). These are associated with the pathway. Conversely, negatively correlated metabolites are implicated in energy metabolism and mitochondrial function (Adenosine 3)’,5’-cyclic monophosphate (cAMP) to taurocholate ratio, Cholate to cAMP ratio, Pyruvate to N-acetylneuraminate ratio), amino acid metabolism and its toxic effects (Dodecanedioate levels, Serine levels, 3-methoxytyrosine levels), lipid metabolism and neuroinflammation (Retinol (vitamin A) to linoleoyl-arachidonoyl-glycerol (18:2 to 20:4) ratio, Phenylacetylcarnitine levels) and other related pathways. MR-Egger and MR-PRESSO tests (**Supplementary Table S8**) showed that there is no horizontal pleiotropy (P>0.05). Furthermore, no obvious heterogeneity was found according to results from Cochrane’s Q test (**Supplementary Table S9**) (P>0.05). The scatter plots for the causal relationship between metabolites and DD were presented in **Supplementary Table S10**. The results from the leave-one-out analysis showed that no individual SNP had a disproportionate effect on the causal estimates. (**Supplementary Tables S11**, **S12**, **Supplementary Figure S4**).

The results of the MR pathway enrichment indicated that the metabolism of glutathione (p < 0.001), glycine, serine and threonine (p < 0.001) and glyoxylate and dicarboxylate (p < 0.001) were all significantly enriched.) were significantly enriched in causally linked metabolites (**Figures 4C, D**) and had a high impact. This finding suggests that these metabolic pathways play a central role in the metabolic regulation of depression and may contribute to the development of depression through multiple mechanisms affecting the redox state, energy metabolism and neurotransmitter synthesis in the nervous system. Furthermore, pathways such as Arginine and proline metabolism, Alanine, aspartate and glutamate metabolism also demonstrated significant enrichment, thereby further substantiating the pivotal role of amino acid metabolism in the mechanism of depression.

### 3.5 Metabolic pathway integration analysis

After excluding metabolites that could not be matched with the KEGG database, A pool of depression-related high-confidence metabolites (n=106) was constructed by integrating LC/GC-MS differential metabolites (n=59), WGCNA hub metabolites (n=20) and MR causal metabolites (n=27). (**Supplementary Table S12**) The results showed that several metabolic pathways were significantly enriched, among which amino acid metabolism, lipid metabolism, nucleotide metabolism and energy metabolism-related pathways were dominant. KEGG enrichment analysis revealed Glycine, Serine and Threonine Metabolism(Pathway-p<0.001;Enrichment-p<0.001), Cysteine and methionine metabolism(Pathway-p<0.001;Enrichment-p<0.001), Alanine, aspartate and glutamate metabolism(Pathway-p<0.001;Enrichment-p<0.001), Purine Metabolism(Pathway-p<0.001;Enrichment-p<0.001)and Glycerophospholipid Metabolism(Pathway-p<0.001)are the core dysregulation pathway (**Figure 4E**). As demonstrated by pathway impact analysis, all of the aforementioned pathways (Impact > 0.2) occupy a pivotal position in the metabolic network (**Figure 4F**). Collectively, these pathways constitute a multidimensional metabolic disorder framework for depression by mediating ATP biosynthesis, regulating redox homeostasis, and influencing membrane lipid dynamics.

This study identified 19 significantly associated metabolic pathways, and 12 pathways were classified into three levels after screening according to the pathway classification criteria (**Table 2**).

**Table 2:** Classification table of metabolic pathways.

Level	Super class	Pathway	Hit Metabolite Count	Pathway analysis p-value	Enrichment analysis p-value	Impact	KEGG ID	LC/GC-MS Results	MR Results
I	Amino Acid Metabolism	Glycine, serine and threonine metabolism	8	<0.01	<0.01	0.29	C00065,C00114,C02291,C01005,C00258,C00097,C03508,C00022	↑	↓
I	Amino Acid Metabolism	Cysteine and methionine metabolism	5	<0.01	<0.01	0.30	C02291,C00065,C00097,C01005,C00022	↑	↓
II	Amino Acid Metabolism	Alanine, aspartate and glutamate metabolism	4	<0.01	<0.01	0.20	C00041,C00025,C00022,C00042	↓	↓
II	Lipid Metabolism	Glycerophospholipid metabolism	4	0.01	0.14	0.22	C00157,C04230,C00114,C00093	↓	↓
II	Nucleotide Metabolism	Purine metabolism	4	0.01	<0.01	0.21	C00385,C00020,C00130,C00387	↑	/
III	Amino Acid Metabolism	Glutathione metabolism	4	<0.01	<0.01	0.04	C00097,C00025 C01879 C00315	↑	↑
III	Vitamin and Cofactor Metabolism	One carbon pool by folate	4	<0.01	0.15	0.06	C00114,C00065,C02291,C00097	↑	↓
III	Carbohydrate Metabolism	Butanoate metabolism	2	0.05	0.04	0	C00025,C00042	↓	↑
III	Lipid Metabolism	Sphingolipid metabolism	4	<0.01	<0.01	0.06	C06124,C00319,C00065,C12144	↓	/
III	Amino Acid Metabolism	Arginine and proline metabolism	3	<0.01	<0.01	0.07	C00315,C00025,C00022	/	↑
III*	Lipid Metabolism	alpha-Linolenic acid metabolism	2	0.01	0.03	0.33	C00157,C06427	/	/
III*	Carbohydrate Metabolism	Glyoxylate and dicarboxylate metabolism	4	<0.01	<0.01	0.09	C02557,C00065,C00025,C00258,C00022	↑	/

*Pathways were Classified into I,II,III categories strictly according to the Classification criteria in the study. III* represents the paradoxical phenomenon of the expression of metabolites contained in this pathway, that is, the rise and fall simultaneously. KEGG IDs were obtained from the KEGG database.

The LC/GC-MS Results ↑ indicated that the expression of metabolites was increased ↓ and decreased expression of metabolites. In MR Results, ↑ it represents a positive correlation with metabolites, and vice versa ↓ represents a negative correlation;/represents no relevant trend was found.

Among the Class I pathways, Glycine, Serine and Threonine Metabolism and Cysteine and Methionine Metabolism showed the most significant regulatory features (Impact ≥ 0.25). Among them, Glycine, Serine and Threonine Metabolism were significantly analyzed in LC/GC-MS, WGCNA, MR and highly correlated pathway pools, and interestingly, its metabolites showed a bidirectional change: a decrease in the protective metabolites Serine and Choline were suggestive of an impaired single-carbon cycle, whereas an elevation in the risky metabolite L- Cysteine was elevated suggesting a compensatory imbalance of oxidative stress. Cysteine and Methionine Metabolism further validated the potential association between the accumulation of Phosphoserine levels and disturbed DNA methylation.

Among the Class II pathways, Alanine, Aspartate and Glutamate Metabolism and Glycerophospholipid Metabolism had Impact values of 0.197 and 0.218, respectively, and the MR analyses showed that their metabolites were significantly associated with depression risk. It is worth noting that Purine Metabolism, although with an Impact value of 0.208, was temporarily Classified as Class II due to the lack of clear causal direction in the MR results, and its accumulation of AMP and Xanthine suggested that there might be a problem with mitochondrial energy.

Among the Class III pathways, Glutathione metabolism, One carbon pool by folate and Butanoate Metabolism had Impact values below 0.1, but the metabolite trends were not contradictory. The rest of the pathways all had ambivalence in metabolite trends. Among them, alpha-linolenic Acid Metabolism was Classified as an ambivalent pathway due to Impact=0.333 but contradictory metabolite trends (decrease in alpha-linolenic acid and increase in Phosphatidylcholine), which may be related to the complex regulation of lipid dynamic balance in depression. Moreover, Glyoxylate and dicarboxylate metabolism with metabolites with elevated risk (Methylmalonyl-CoA, Glutamic acid) and reduced risk (Serine, Pyruvic acid) were Classified as contradictory pathways.

Further pathway impact analysis indicated that the Class I and II pathways form a functional interplay network through glycine, purine, and sphingolipid metabolism (**Figure 4F**). These three processes jointly regulate the three core biological processes of the methylation cycle, membrane lipid homeostasis and energy metabolism. This pathway hierarchical framework provides a theoretical basis for metabolic typing and targeted intervention in depression.

## 4 Discussion

Several studies have reported the presence of metabolic pathway disorders in patients with DD in comparison to the healthy population. In this study, we identified key metabolic disruptions in elderly patients with depression by integrating non-targeted metabolomics, WGCNA modular analysis, and MR methods. Utilizing the enrichment and influence of metabolic pathways and the consistency of multi-omics, a three-tiered framework of metabolic regulation in depression (Class I-III pathways) was constructed, providing a novel perspective on the mechanism of the core metabolic pathways and potential intervention targets. Among the pathways identified, significant alterations were observed in amino acid and lipid metabolism, with particular emphasis on disruptions related to glycine-serine-threonine metabolism and cysteine-methionine metabolism. These pathways may reflect the disturbance of one-carbon metabolism and the oxidative stress response, though further validation is needed to confirm their direct impact. Interestingly, phosphoserine accumulation was noted, which may relate to alterations in DNA methylation processes, highlighting a possible role of epigenetic regulation in depression.

In contrast to earlier studies, which have largely focused on the tryptophan/kynurenine axis, the present study reveals specific alterations in serine metabolic pathways, such as phosphorylation modifications, in elderly depressed individuals. This discrepancy may reflect the distinct metabolic characteristics of this population. Moreover, the elevated levels of L-cysteine observed suggest a compensatory response in glutathione synthesis, indicating that redox imbalances may play a significant role in geriatric depression.

### 4.1 Cross-omics driven hierarchical core metabolic

The Class I pathways screened in this study (Glycine, serine and threonine metabolism; Cysteine and methionine metabolism) exhibited the highest pathway impact (Impact ≥ 0.25) in metabolic disorders of depression. This is consistent with previous findings (24). Serum choline levels in the Glycine-serine-threonine metabolic axis are associated with an increased risk of depression and positively correlate with depressive-like behaviors in animal models (25). However, Serine was significantly reduced in the present study along with Choline, and such a phenomenon may be related to impaired single-carbon cycling (26).

At the same time, LCysteine was abnormally elevated, possibly related to an imbalance in oxidative stress compensation (27). This pattern of cysteine accumulation may suggest a disrupted redox balance, which is commonly observed in depression. In the Cysteine-methionine metabolic pathway, L Cystathionine homeostasis is associated with the vitamin B6-dependent transsulfuration pathway, which is one of the significant pathways of homocysteine metabolism (28). Stagnation in this pathway could contribute to neurotoxic effects associated with homocysteine accumulation, although further research is required to confirm these potential relationships (29). Although the two pathways present partial contradictions in metabolite trends, they collectively point to methyl donor depletion and redox imbalance triggered by mitochondrial dysfunction (30). This suggests that targeting the single-carbon cycle may be a key entry point for intervening in metabolic disorders in depression.

### 4.2 Dynamically reconstituted metabolic sub-networks with functional complementarity

Class II pathways (Alanine-aspartate-glutamate metabolism; Glycerophospholipid metabolism; Purine metabolism), although slightly lower in impact (0.1 ≤ Impact < 0.25), yet their alterations suggest a complex regulatory network involving neurotransmitter imbalances in depression. Specifically, increased glutamic acid levels may disrupt central nervous system function, potentially through impaired blood-brain barrier integrity or NMDA receptor activation, which in turn could trigger mitochondrial dysfunction. Additionally, glutamate-induced neuroinflammation has been suggested as a contributing factor to depression, highlighting the potential role of neuroinflammatory pathways in the disorder’s pathophysiology (31). This is consistent with imaging findings of hyperglutaminergic signaling in the prefrontal cortex in depression (32). In the glycerophospholipid metabolism pathway, supplementation with phosphatidylcholine in animal models has been shown to ameliorate depression-like behaviors, such as reduced resting time in the forced swim test, improved spatial cognition, and enhanced hippocampal neurogenesis (33). Accumulation of AMP/Xanthine in Purine metabolism suggests reduced mitochondrial ATP synthesis, (34) a phenomenon that may be mechanistically related to the reduction of mitochondrial DNA copy number in peripheral blood in depressed patients, suggesting a possible dysfunction in mitochondrial energy production. However, there is some heterogeneity in the mentioned pathways in terms of the directionality of metabolite changes, in that a decrease in energy metabolism-related metabolites coexists with abnormal fluctuations in neurotransmitter precursors, which may be related to the sample of the DD population included in the present study (GDS=14.71 ± 3.57 for mild depression). This heterogeneity may be related to a dysregulation of mitochondrial-endoplasmic reticulum coupling, disrupting synaptic plasticity and inflammatory homeostasis (35). It may also be because different subtypes or stages of depression have specific metabolic profiles (36).

### 4.3 Contradictory regulation of heterogeneous metabolic nodes: a multi-omics paradox

Class III pathways (Glutathione metabolism, One carbon pool by folate) were statistically significant. Although the influence of the pathways was weaker, the observed changes in these pathways suggest possible involvement in oxidative stress regulation in depression. Inverse changes in glutathione metabolism of L-cysteine and Pyroglutamic acid may reflect compensatory activation of the γ-glutamyl cycle in response to chronic oxidative stress, with elevated L-cysteine supporting glutathione synthesis and decreased Pyroglutamic acid indicating reduced glutathione degradation in response to oxidative damage, which may be a possible. These findings may point to potential self-regulatory mechanisms in individuals with mild depression (37).

In the paradoxical pathway, α-linolenic acid metabolism shows paradoxical changes in phosphatidylcholine and α-linolenic acid, which may be related to the compartmentalized regulation of ω-3 fatty acid metabolism (38). In the presence of an ω-3 deficiency in the CNS due to a limitation of the blood-brain barrier, peripheral adipose tissue lipolysis is triggered by vagally mediated hepatic-brain axis signaling, (39) leading to a decrease in plasma. This leads to a compensatory increase in plasma-free α-linolenic acid. This compensatory mechanism temporarily alleviates ω-3 deficiency in the brain. However, it does not reverse the impaired phospholipid remodeling of neuronal membranes, which ultimately leads to an increase in the synthesis of proinflammatory prostaglandins (e.g., PGE2) (40).These paradoxical phenomena, which may stem from heterogeneity in metabolic regulation or a lagged plasma response to changes in central metabolism, must be further validated with multi-omics techniques and large population samples.

### 4.4 Integrated model of the multidimensional metabolic regulatory interface

The systematic regulatory network of peripheral metabolic disorders in elderly patients with depression has been systematically revealed through the inferential integration and analysis of the above results. These results suggest a dynamic, interactive process in which oxidative stress and methylation imbalances may act as central drivers of depression. Specifically, abnormal glutathione metabolism and impaired single-carbon cycling may reduce the capacity for reactive oxygen species (ROS) scavenging and inhibit DNA methyltransferase activity, contributing to a state of genome-wide hypomethylation Single-carbon metabolism, particularly its role in methylation processes, could be central to understanding these disruptions (41, 42).

Additionally, elevated levels of cysteine and changes in phosphatidylcholine metabolism were observed, which may reflect attempts by the body to compensate for oxidative stress. These findings suggest that lipid metabolism and redox balance are important contributors to the metabolic dysfunction observed in depression, warranting further research into how lipid metabolism may influence neuroinflammation and synaptic plasticity in this context (43). The study also uncovered disruptions in purine metabolism, particularly the accumulation of AMP and xanthine, pointing to potential disturbances in mitochondrial ATP synthesis. These findings support the role of mitochondrial dysfunction in depression but suggest that further investigation is needed to explore the relationship between mitochondrial energy production and ATP synthesis in the depressive state (44).

In summary, this study highlights the significant role of single-carbon metabolism and oxidative stress in the metabolic dysfunction associated with depression. Future research should focus on understanding how these disruptions contribute to the underlying pathophysiology of depression and explore potential therapeutic targets aimed at restoring single-carbon metabolism, mitochondrial function, and lipid homeostasis to mitigate depressive symptoms (45, 46).

### 4.5 Innovation and limitations

In this study, we propose a novel approach to address the limitations of conventional single-omics studies by integrating WGCNA modular analysis, MR causal inference, and cross-platform metabolite validation. This approach enables the revelation of the core regulatory status of multiple metabolic pathways in geriatric depression, with a focus on single-carbon cycle metabolism and methylation imbalance. Constructing a three-level analysis framework comprising “metabolomics - network module - causal inference” enabled the combined analysis of metabolites. The identification of key metabolites associated with nanocarbon cycle metabolism and methylation imbalance has enabled a comprehensive exploration of the multidimensional metabolic dysregulation phenomenon in depression. Furthermore, a substantial sample size from a Chinese community-based elderly population addresses a significant gap in studying metabolic markers and pathways in DD within Asian populations.

However, further exploration is necessary to address the following limitations: plasma metabolites are challenging to fully reflect the dynamic changes *in vivo*, and they may be inadequate for detecting depression-related metabolites. Additionally, although the WGCNA method is suitable for metabolite clustering detection, the number of significant metabolites detected by WGCNA in this study is relatively small, which suggests that the results may have certain limitations. It is important to note that the Classification system presented here serves only as a summary of the analytical standards for the results of this study and should not be directly applied as a tiered Classification system. Doing so could potentially overestimate its biological interpretation. Furthermore, the thresholds used in the Classification system may not fully capture the complexity of metabolic pathways. Given the intricate and multi-dimensional nature of the GC-MS data, overfitting remains a concern. The complexity of the underlying biological mechanisms, particularly the body’s regulatory responses to depression, might result in the model focusing on noise or irrelevant patterns, rather than generalizable relationships. Despite these efforts, we acknowledge that further studies with larger, more diverse cohorts, as well as more refined methodological approaches, are needed to fully validate these findings.

## 5 Conclusion

In this study, we integrated untargeted metabolomics, WGCNA and MR methods to systematically reveal the hierarchical disordered characteristics of plasma metabolic networks in elderly patients with depression. We constructed the first metabolic pathway Classification framework based on multi-omics data. We identified 1,458 plasma metabolites by LC/GC-MS platform, screened 20 pivotal metabolites by combining with WGCNA, and confirmed 29 metabolites were causally associated with depression by MR analysis.

The study found significant alterations in glycine-serine-threonine metabolism and cysteine-methionine metabolism, which are linked to oxidative stress and single-carbon metabolism. Elevated cysteine levels suggest a compensatory response to oxidative stress, while disruptions in purine metabolism point to possible mitochondrial dysfunction, a key factor in energy production. Notably, our results indicate that single-carbon metabolism, particularly its role in DNA methylation, may play a central role in depression. Serine deficiency and phosphoserine accumulation could contribute to genome-wide hypomethylation, providing new insights into the potential epigenetic mechanisms involved in depression.

These findings emphasize the importance of oxidative stress and metabolic dysfunction in depression. Future research should explore the therapeutic potential of targeting single-carbon metabolism, mitochondrial function, and lipid imbalances as strategies for treating depression, particularly in older adults. Meanwhile, these results provide new possible directions for metabolic typing and intervention in depression and suggest that future studies should focus on the validation of different populations, the dynamic changes of metabolic markers, and the integration of multi-omics data to promote the development of precision medicine.

## Data Availability

The raw data supporting the conclusions of this article will be made available by the authors, without undue reservation.
