# Supplementary material for: Multi-omics investigation of metabolic dysregulation in depression: integrating metabolomics, weighted gene co-expression network analysis, and mendelian randomization
Source: Front Psychiatry. 2025 Aug 12;16:1627020. doi: 10.3389/fpsyt.2025.1627020 (PMC12378940; doi:10.3389/fpsyt.2025.1627020)
Supplement: Supplementary file 1 [file Supplementaryfile1.docx]

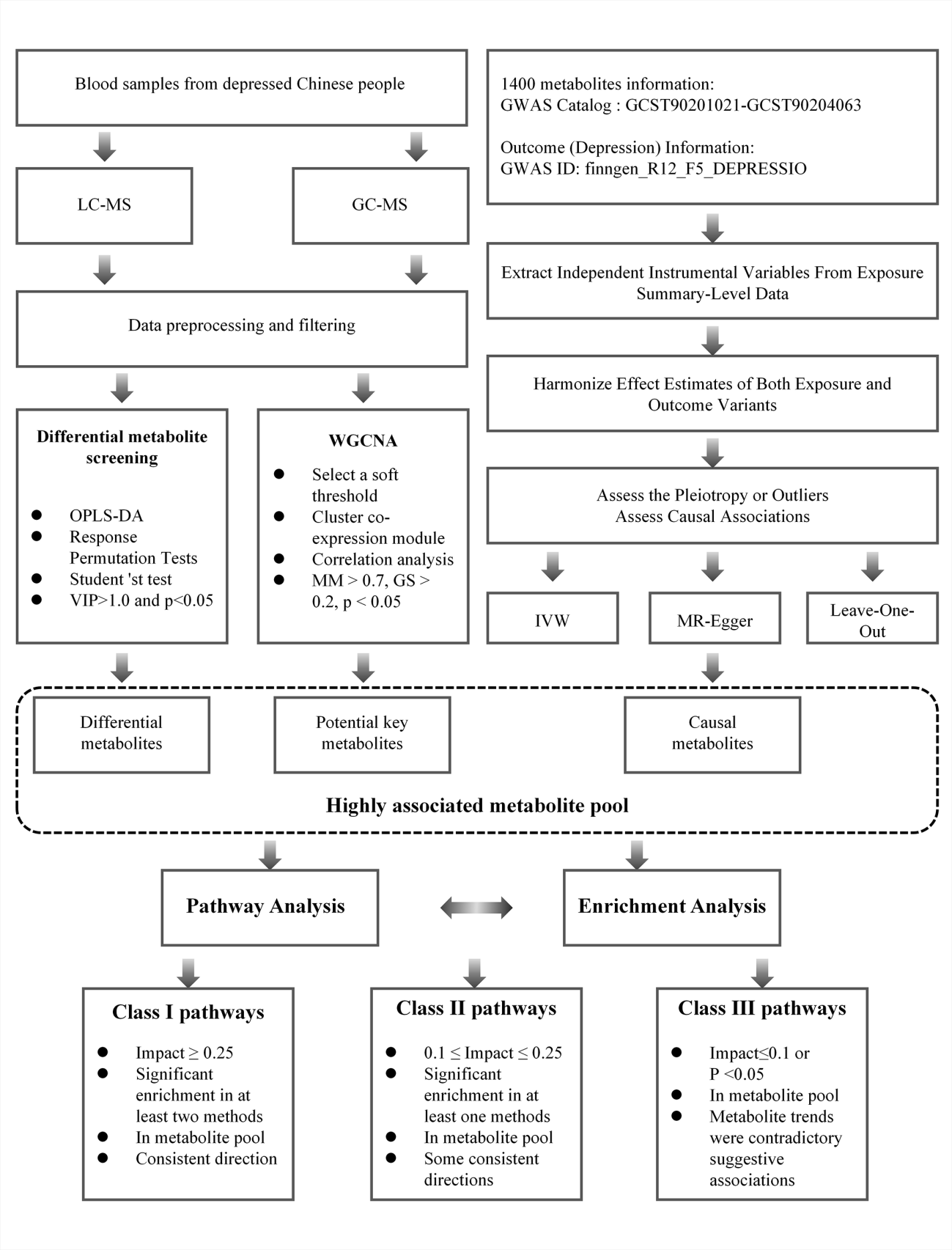


**Figure S1:** Study design flow chart.


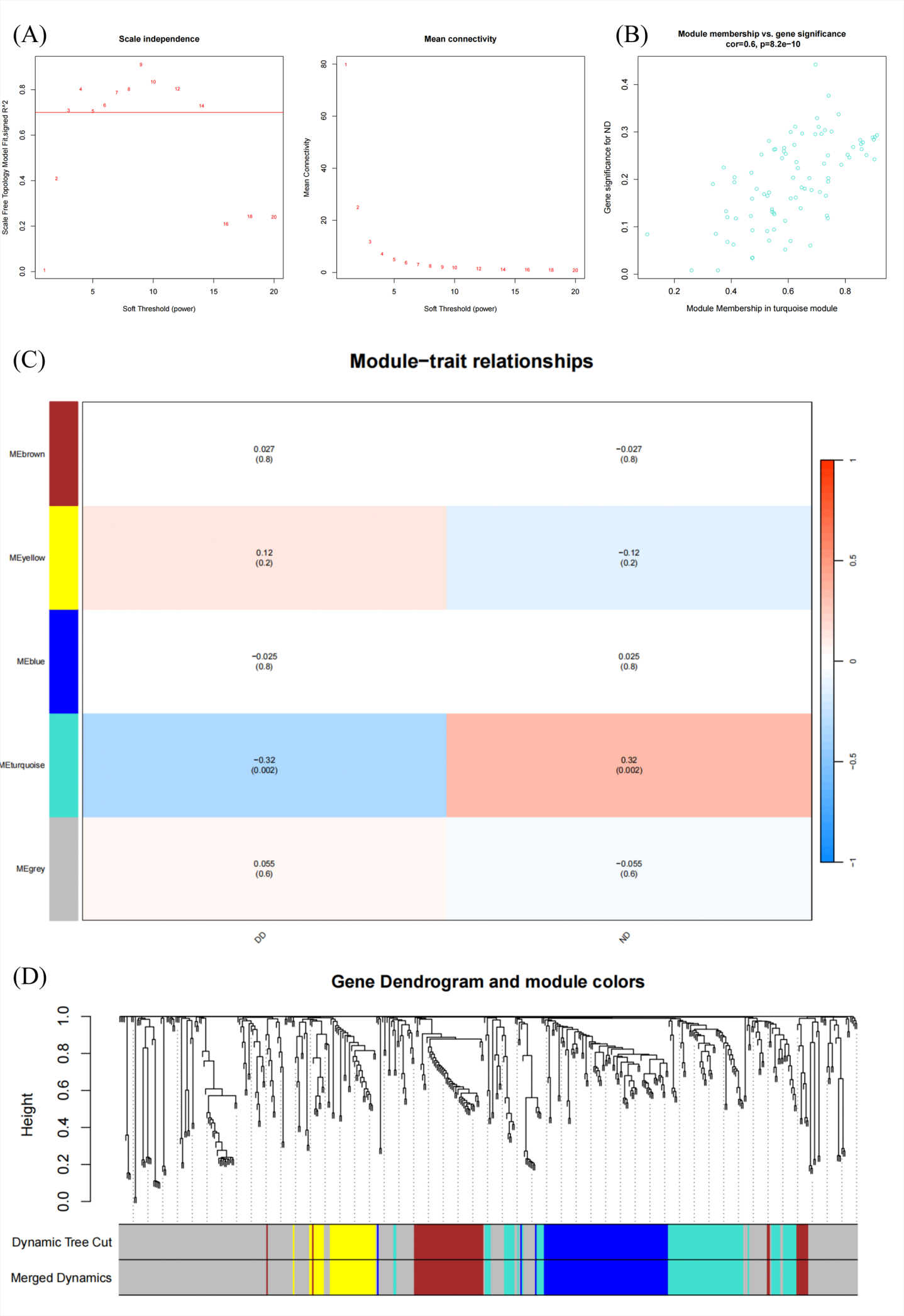


**Figure S2:** LC-MS-based implementation of WGCNA for differential metabolites and identification of key module metabolites. (A) The soft threshold (β) for LC-MS-based differential metabolites was set to 5, ensuring a scale-free topology with an R² value of 0.7. (B) The Scatterplot for LC-MS-based differential metabolites in the turquoise module shows the relationship between module membership and metabolite importance. Higher module membership values indicate a stronger association with the turquoise module. (C)Comparison of module-trait relationships between the ND and DD groups based on LC-MS differential metabolites. Each module is color-coded to indicate its correlation with the traits of interest. (D) Clustering dendrogram of coexpression network modules from WGCNA based on LC-MS differential metabolites, showing hierarchical clustering of modules as a function of heterogeneity measure.

**
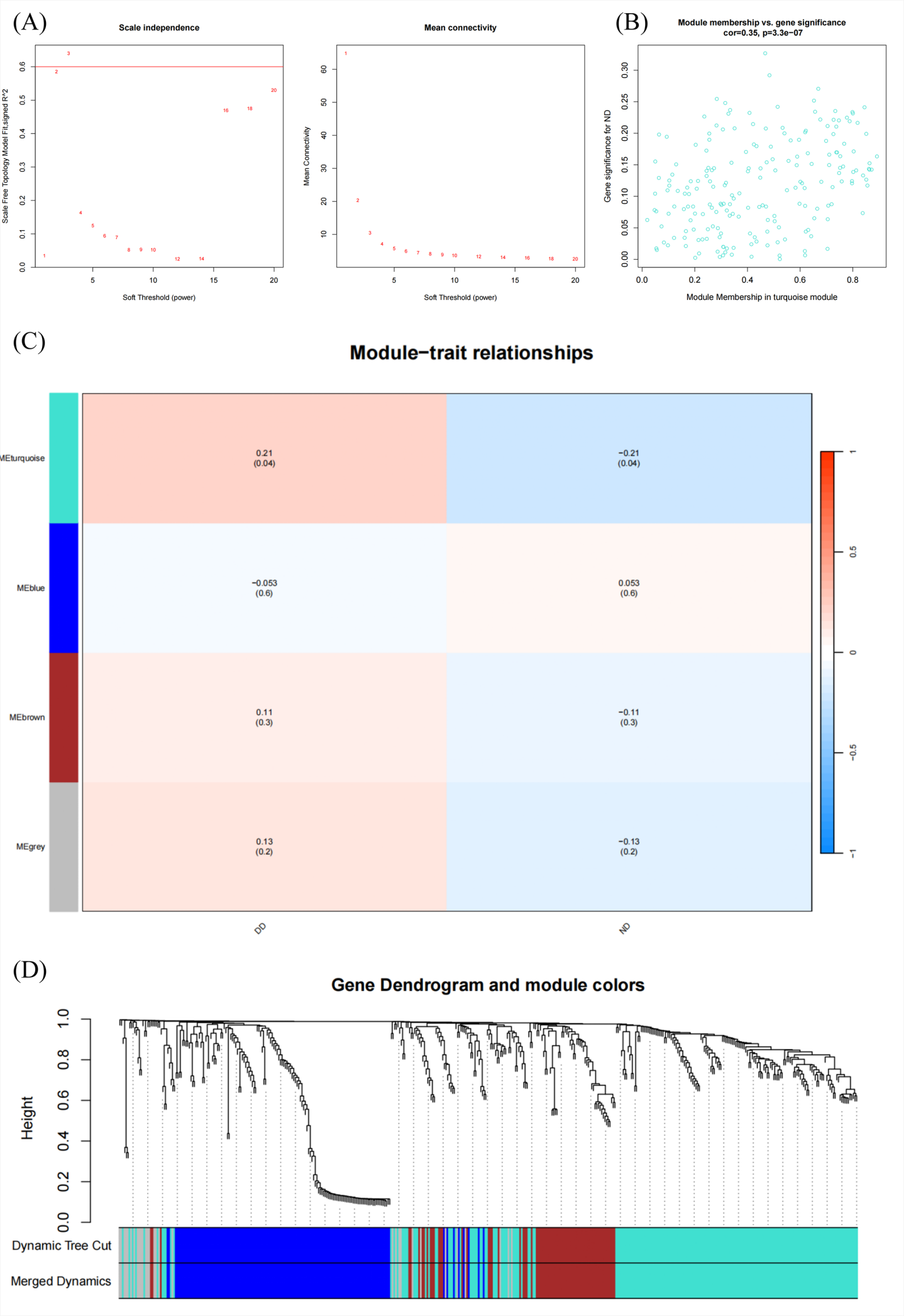
**

**Figure S3:** GC-MS-based implementation of WGCNA for differential metabolites and identification of key module metabolites. (A) The soft threshold (β) for GC-MS-based differential metabolites was set to 3, ensuring a scale-free topology with an R² value of 0.6. (B) The scatterplot for GC-MS-based differential metabolites in the turquoise module shows the relationship between module membership and metabolite importance. (C) Comparison of module-trait relationships between the ND and DD groups based on GC-MS differential metabolites, with similar color coding for module-trait correlations. (D) Clustering dendrogram of coexpression network modules from WGCNA based on GC-MS differential metabolites, showing hierarchical clustering as a function of heterogeneity measure.


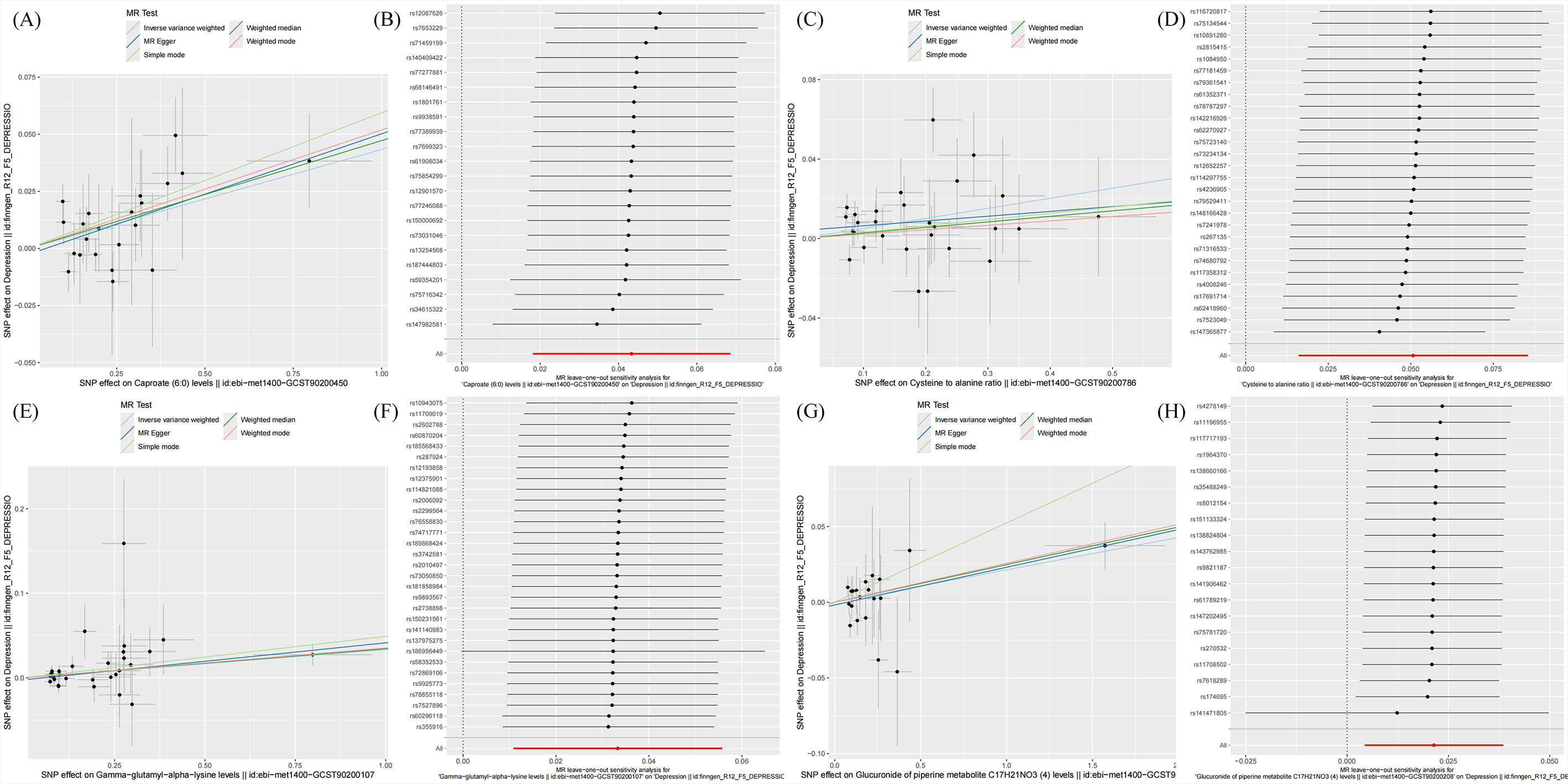


**Figure S4:** Scatter plots and Leave-one-out plots for the high association between metabolites and depression. (A, B): GCST90200450: Caproate (6:0) levels; C, D): GCST90200786: Cysteine to alanine ratio; (E, F): GCST90200107: Gamma-glutamyl-alpha-lysine levels; (G,H): GCST90200208: Glucuronide of piperine metabolite C17H21NO3 (4) levels.


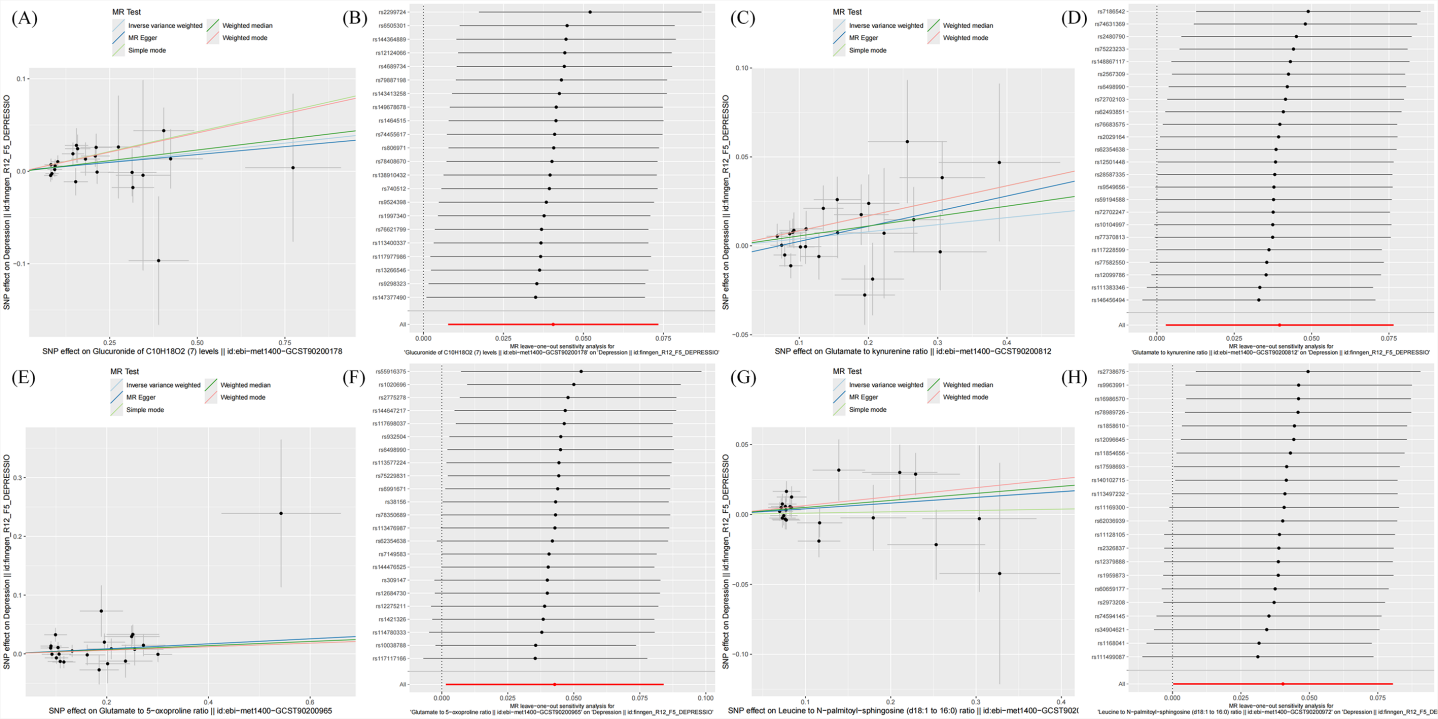


**Figure S5:** Scatter plots and Leave-one-out plots for the high association between metabolites and depression. (A, B): GCST90200178: Glucuronide of C10H18O2 (7) levels; (C, D): GCST90200812: Glutamate to kynurenine ratio; (E, F): GCST90200965: Glutamate to 5-oxoproline ratio; (G,H): GCST90200972: Leucine to N-palmitoyl-sphingosine (d18:1 to 16:0) ratio.


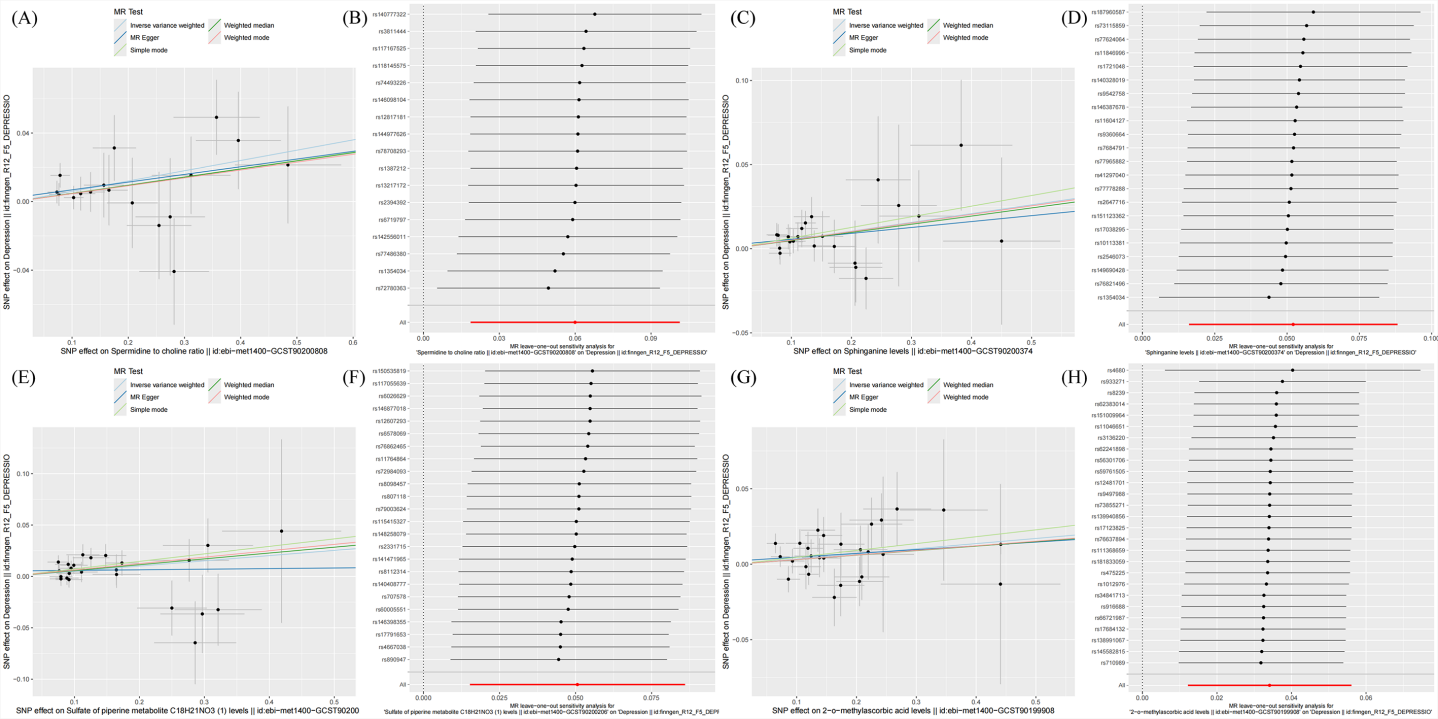


**Figure S6:** Scatter plots and Leave-one-out plots for the high association between metabolites and depression. (A, B): GCST90200808: Spermidine to choline ratio; (C, D): GCST90200374: Sphinganine levels; (E, F): GCST90200206: Sulfate of piperine metabolite C18H21NO3 (1) levels; (G,H): GCST90199908: 2-o-methylascorbic acid levels.


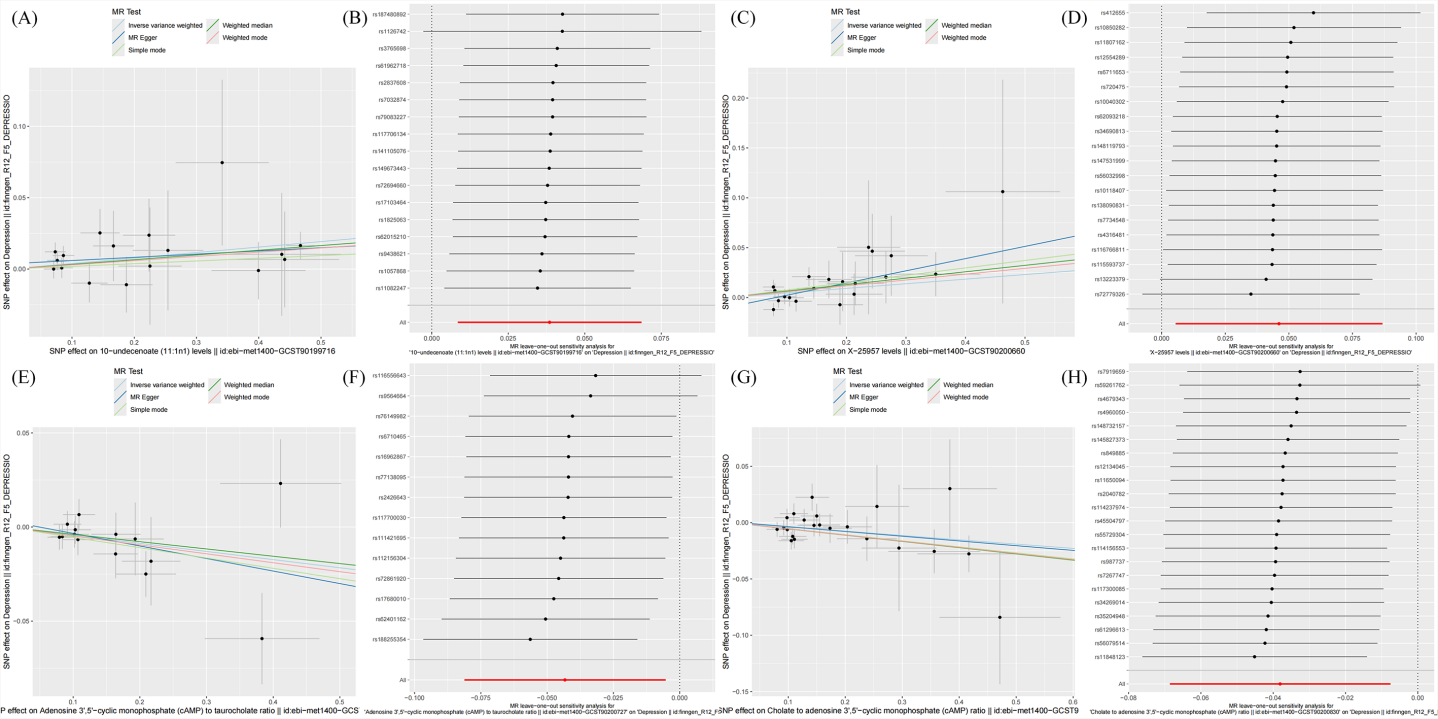


**Figure S7:** Scatter plots and Leave-one-out plots for the high association between metabolites and depression. (A, B): GCST90199716: 10-undecenoate (11:1n1) levels; (C, D): GCST90200660: X-25957 levels; (E, F): GCST90200727: Adenosine 3',5'-cyclic monophosphate (cAMP) to taurocholate ratio; (G,H): GCST90200830: Cholate to adenosine 3',5'-cyclic monophosphate (cAMP) ratio.


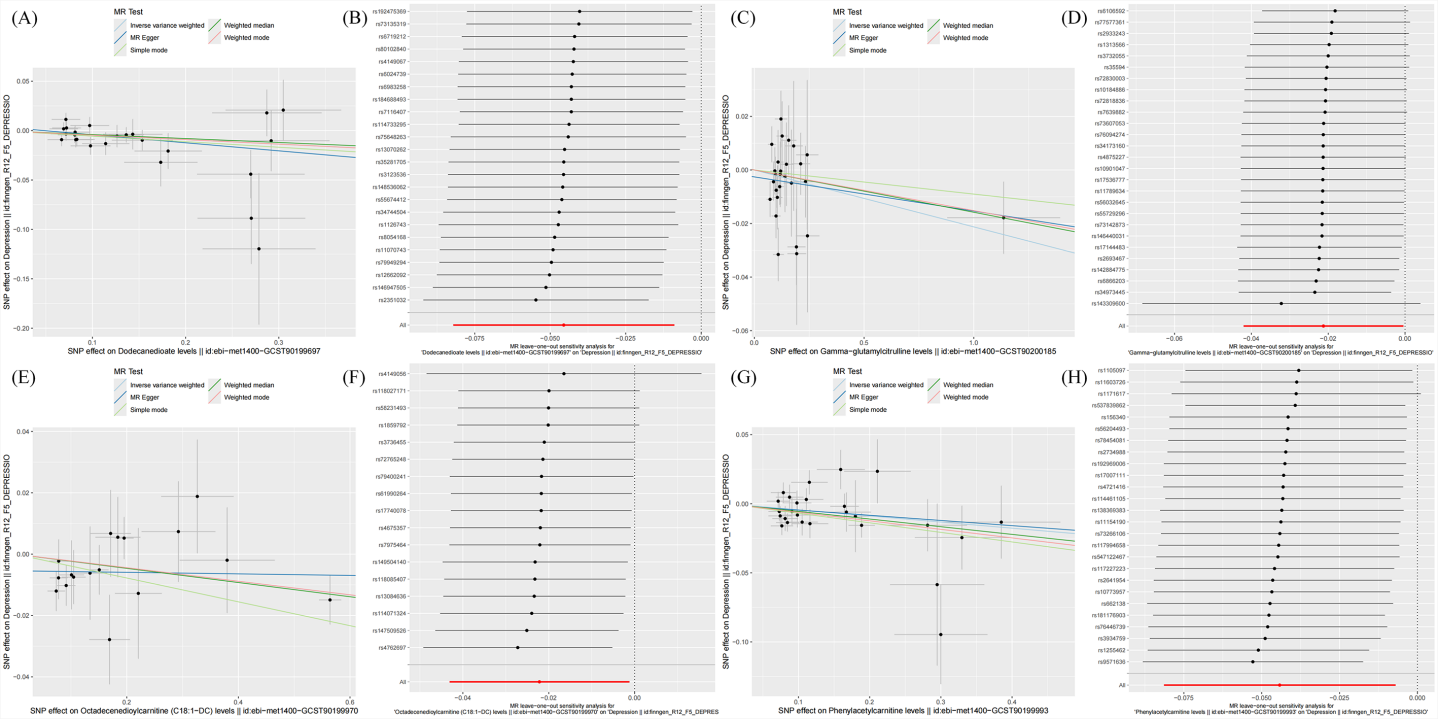


**Figure S8:** Scatter plots and Leave-one-out plots for the high association between metabolites and depression. (A, B): GCST90199697: Dodecanedioate levels; (C, D): GCST90200185: Gamma-glutamylcitrulline levels; (E, F): GCST90199970: Octadecenedioylcarnitine (C18:1-DC) levels; (G,H): GCST90199993: Phenylacetylcarnitine levels.


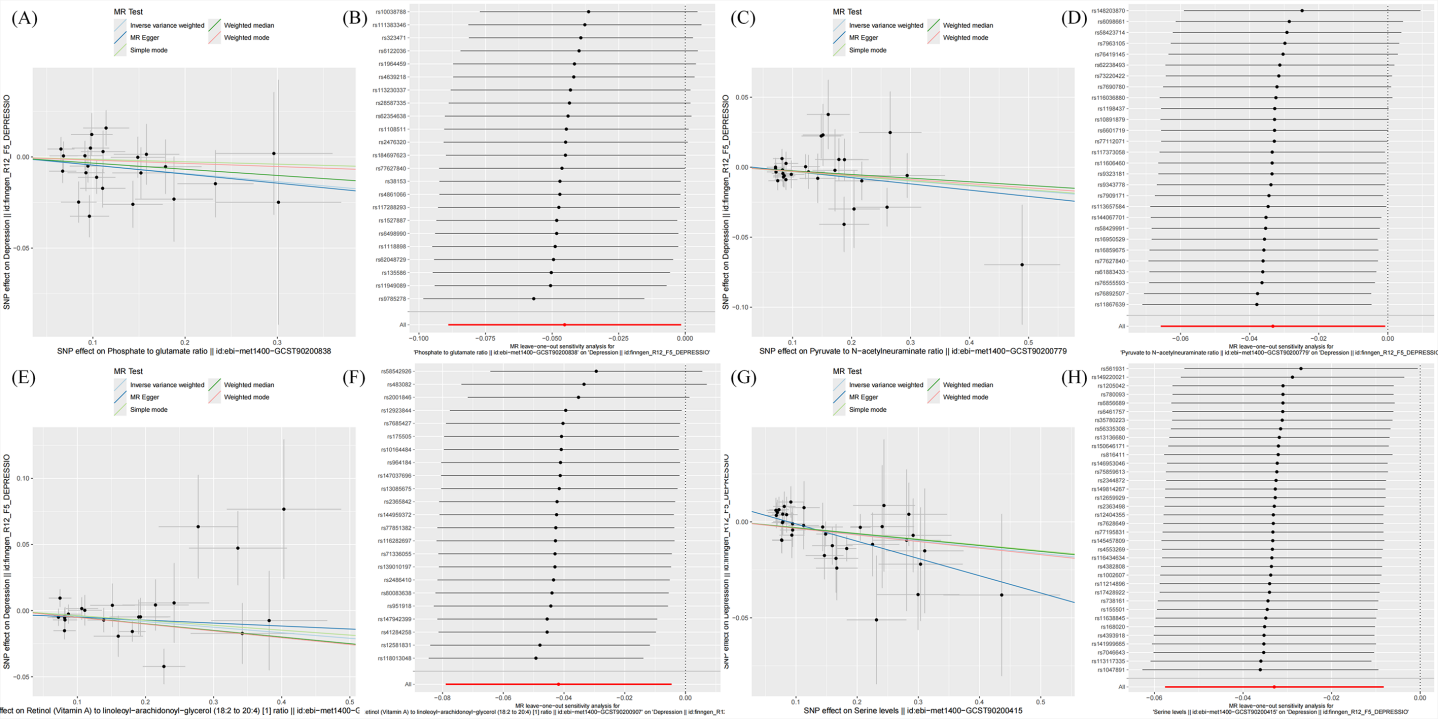


**Figure S9:** Scatter plots and Leave-one-out plots for the high association between metabolites and depression. (A, B): GCST90200838: Phosphate to glutamate ratio; (C, D): GCST90200907: Retinol (Vitamin A) to linoleoyl-arachidonoyl-glycerol (18:2 to 20:4) ratio; (E, F): GCST90199970: Octadecenedioylcarnitine (C18:1-DC) levels; (G,H): GCST90200415: Serine levels.


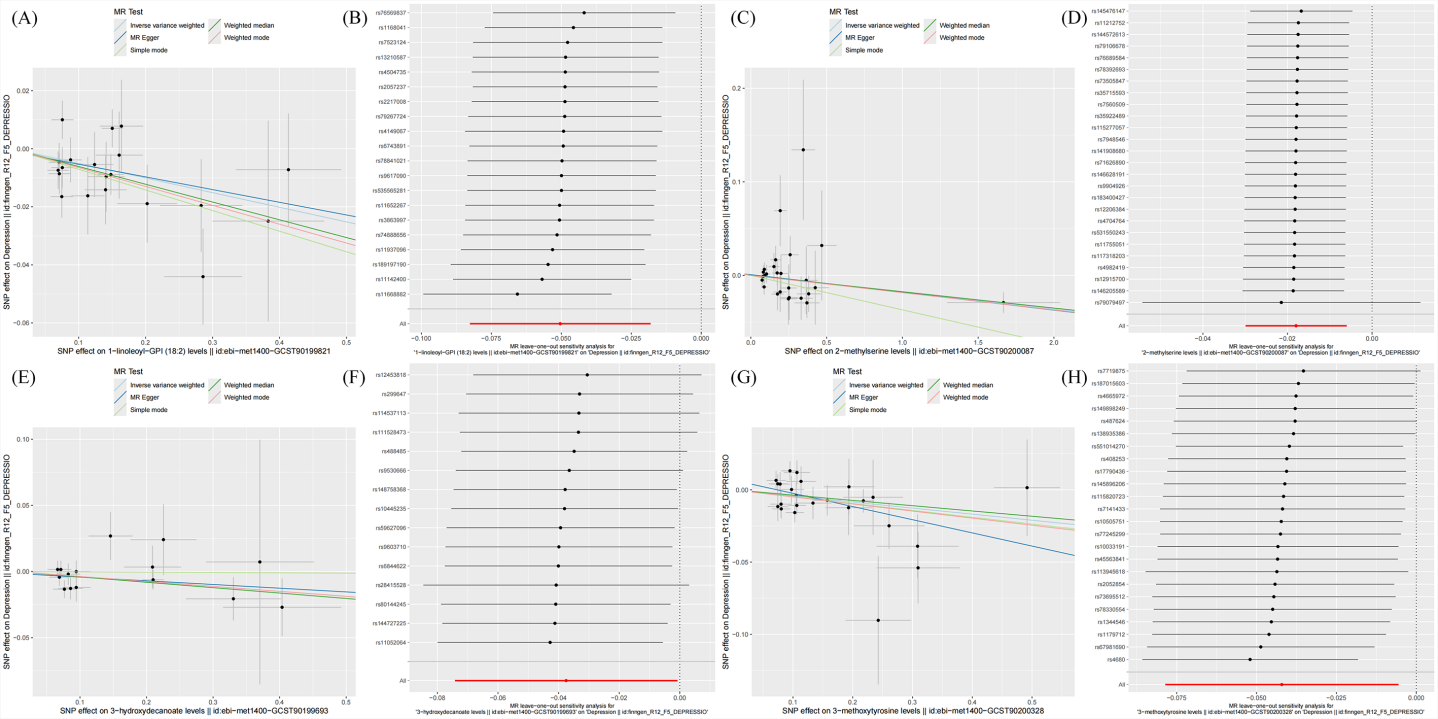


**Figure S10:** Scatter plots and Leave-one-out plots for the high association between metabolites and depression. (A, B): GCST90199821: 1-linoleoyl-GPI (18:2) level; (C, D): GCST90199693: 3-hydroxydecanoate levels; (E, F): GCST90199970: Octadecenedioylcarnitine (C18:1-DC) levels; (G,H): GCST90200328: 3-methoxytyrosine levels.


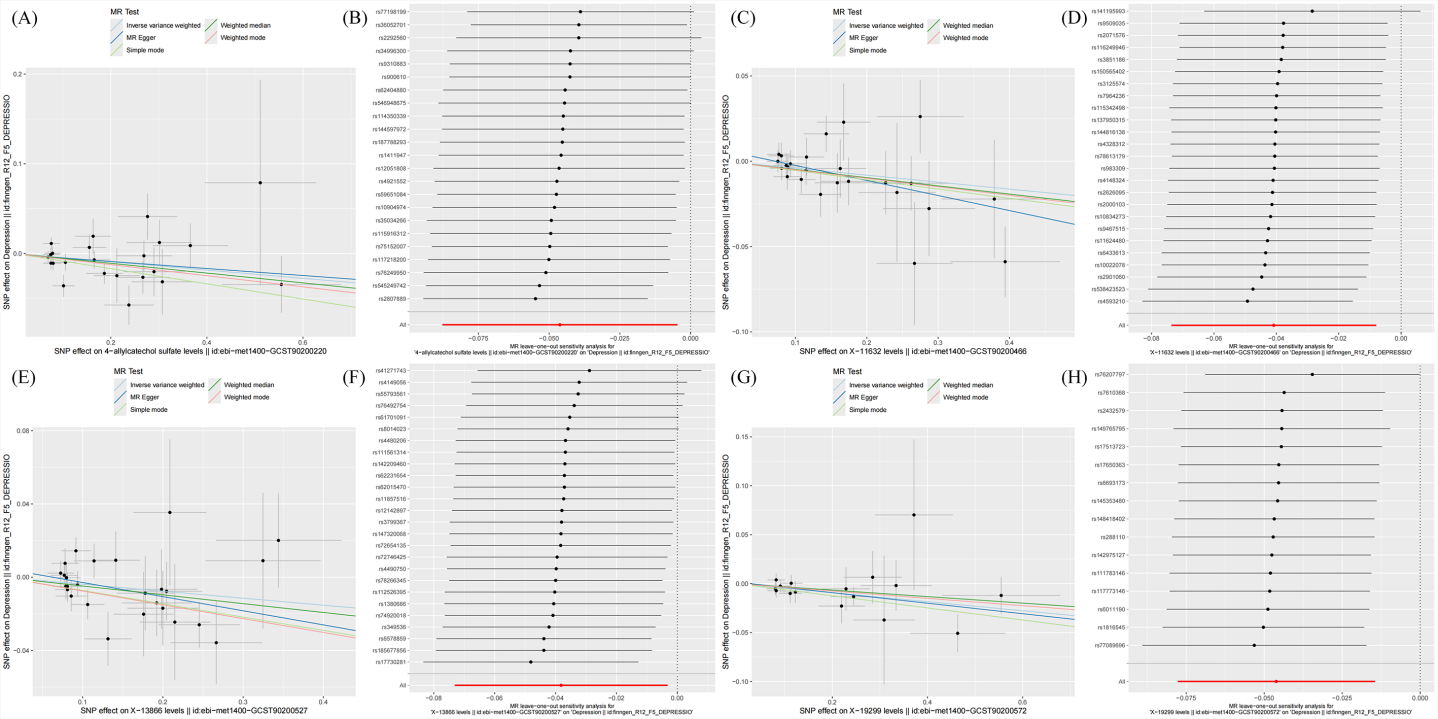


**Figure S11:** Scatter plots and Leave-one-out plots for the high association between metabolites and depression. (A, B): GCST90200220: 4-allylcatechol sulfate levels; (C, D): GCST90200466: X-11632 levels; (E, F): GCST90200527: X-13866 level; (G,H): GCST90200572: X-19299 levels.


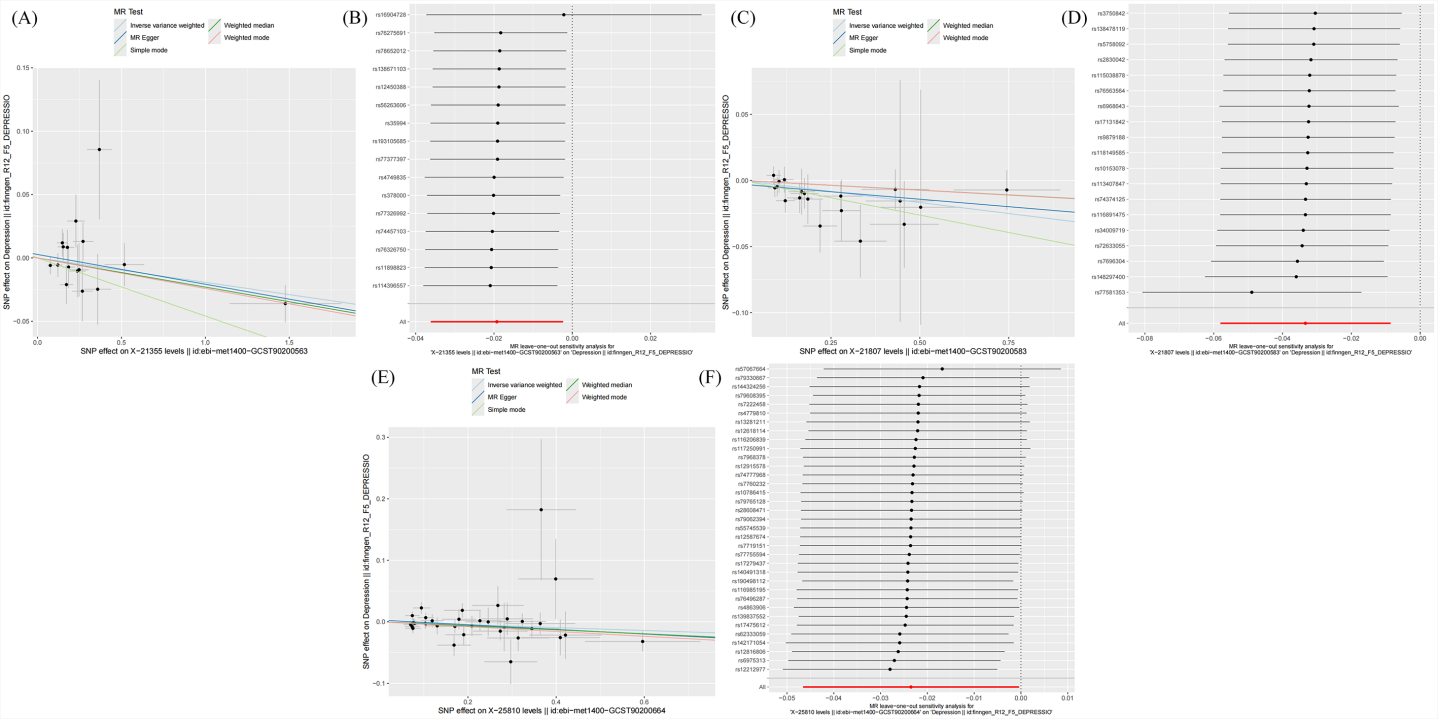


**Figure S12:** Scatter plots and Leave-one-out plots for the high association between metabolites and depression. (A, B): GCST90200563: X-21355 levels; (C, D): GCST90200583: X-21807 levels; (E, F): GCST90200664: X-25810 levels.
